# Comprehensive Discovery of the Accessible Primary Amino Group-Containing Segments from Cell Surface Proteins by Fine-Tuning a High-Throughput Biotinylation Method

**DOI:** 10.3390/ijms24010273

**Published:** 2022-12-23

**Authors:** Tamás Langó, Katalin Kuffa, Gábor Tóth, Lilla Turiák, László Drahos, Gábor E. Tusnády

**Affiliations:** 1 Protein Bioinformatics Research Group, Institute of Enzymology, Research Centre for Natural Sciences, Magyar Tudósok krt 2, H-1117 Budapest, Hungary; 2 Doctoral School of Biology, Institute of Biology, ELTE Eötvös Loránd University, Pázmány P. stny. 1/C, H-1117 Budapest, Hungary; 3 MS Proteomics Research Group, Institute of Organic Chemistry, Research Centre for Natural Sciences, Magyar Tudósok krt 2, H-1117 Budapest, Hungary

**Keywords:** biotinylated peptides, cell surface proteins, affinity enrichment, solid-phase extraction, HPLC, mass spectrometry, surfaceome

## Abstract

Cell surface proteins, including transmembrane and other surface-anchored proteins, play a key role in several critical cellular processes and have a strong diagnostic value. The development of quick and robust experimental methods remains vital for the accurate and comprehensive characterization of the cell surface subproteome of individual cells. Here we present a high-throughput technique which relies on the biotinylation of the accessible primary amino groups in the extracellular segments of the proteins, using HL60 as a model cell line. Several steps of the method have been thoroughly optimized to capture labeled surface proteins selectively and in larger quantities. These include the following: improving the efficiency of the cell surface biotinylation; reducing the endogen protease activity; applying an optimal amount of affinity column and elution steps for labeled peptide enrichment; and examining the effect of various solid-phase extraction methods, different HPLC gradients, and various tandem mass spectrometry settings. Using the optimized workflow, we identified at least 1700 surface-associated individual labeled peptides (~6000–7000 redundant peptides) from the model cell surface in a single nanoHPLC-MS/MS run. The presented method can provide a comprehensive and specific list of the cell surface available protein segments that could be potential targets in various bioinformatics and molecular biology research.

## 1. Introduction

The cell surface markers of the distinct cell types play a central role in different physiological processes. They can be a part of the direct or indirect interactions, such as protein–protein interactions, protein–glycan moieties, and protein–lipid contacts, involved in ion transport mechanisms [1], nutrient acquisition [2], and cell signaling [3]. Currently, nearly two-thirds of approved human drug targets are cell surface proteins (CSPs) based on the DrugBank database [4,5]. According to their importance, the detailed characterization of cell surface accessible segments is crucial for targeted therapy, such as immunotherapy [6] or the development of vaccines against particular infectious diseases [7]. Nevertheless, due to their relatively low abundance, compared with the other cellular proteins [8,9] and their specific physical-chemical properties (especially in the case of transmembrane proteins [10]), achieving their comprehensive identification and their extracellular accessible segments remains a difficult task.

For individual detection of the CSPs, antibody-based approaches have been traditionally used, including flow cytometry, Western blot, or immunohistochemistry analysis, but their dependence on the available antibody collections is indisputable [11]. To overcome this limitation, many high-throughput methods (mainly with mass spectrometry detection) have been developed for the characterization of CSPs and/or their specific regions [9,12]. These methods can be classified based on enrichment strategies preceding the MS analysis, which include ultracentrifugation to obtain membrane protein fractions [13], enzymatic treatment of cell surface to promote the extracellular peptide release [14], or partial proteolysis of surface proteins to cause new peptide termini that can be labeled and separated based on the modification [10]. Additional strategies are the attachment of cationic colloidal silica beads to the membranes to promote their isolation with increasing density [15] and the use of biotin-containing reagents with various chemical specificities [16,17,18,19]. The latter has been utilized in the current study and is discussed further below.

The vast majority of the chemical modification strategies for CSPs utilize two different pipelines. Some methods target the extracellularly exposed post-translational modifications on the CSPs (as *N*-glycan moieties [20,21]), while other approaches modify the accessible/free side chains of various residues (lysine [10,19] or aspartic and glutamic acids [22]) on the outside region of the plasma membrane. *N*-glycopeptide enrichment offers a highly specific and selective method for the identification of extracellular segments of CSPs [23]; however, the frequency of the N-X-S/T/C motifs is lower than other reactive side chains containing residues [19]. A further disadvantage of this method is that it requires a large amount of starting material (~3 × 10^7^–10^8^ cells per experiment [24]), and it is also difficult to apply for rare cell types or primary cells. Therefore, the residue-specific approaches are more widespread, cysteines and lysines being the most targeted residues for the covalent modifications/biotinylations [25,26]. The main advantage of these methods is that free sulfhydryls and primary amino groups can be labeled in a one-step reaction. In contrast, labeling the carboxyl group of aspartic and glutamic acid residues is a two-step reaction (EDC and Sulfo-NHS pre-activation of carboxyl groups, then biotinylation by biotinyl cystamine [22,27]), and therefore, it is not frequently used.

The best-known primary amino group-specific chemical reagent is Sulfo-NHS-SS-biotin, which is the cornerstone of the developed method in the current study. It is membrane-impermeable; therefore, labeling is only carried out on the extracellular regions of the CSPs. Moreover, it has a cleavable spacer arm (containing a disulfide bridge) promoting the elution of the cell surface biotinylated components from the affinity column using reducing agents (such as DTT, 2-mercaptoethanol or TCEP, although the latter may cause unwanted chemical reactions during the elution that makes the MS analysis more difficult [19]). This is an unquestionable advantage compared with similar reagents with a non-cleavable spacer arm, where the strongest non-covalent biological interaction (between the avidin and the biotin [28], Kd = 10^−15^) should be dissociated by harsh elution conditions [29] or by digestion on the affinity column [30]. These strategies often complicate the subsequent peptide analysis; thus, the cleavable biotinylation of the CSPs is preferred. Sulfo-NHS-SS-biotin is mostly used for complete protein isolation [31,32] but is sometimes also applied for cell surface peptide enrichment strategies [19,22].

Protein-level enrichment methods have several drawbacks. First of all, abundant cytosolic proteins can strongly associate with the modified proteins, and their removal is difficult even after vigorous washing steps [33]. Therefore, the list of identified proteins may contain non-cell surface proteins, thus complicating the exact proteome-wide view of the labelable CSPs from the examined cell surface. Ion suppression by non-labeled peptides during MS analysis (similar to the ubiquitination site identification studies [34]) and incorrectly set database search parameters (modification-specific mass on the targeted residues not set in the used search engine) [35,36] could compromise the detection of the exact enrichment-specific regions of the particular proteins. In addition, the size of the modification can also compromise the identification as in the bioorthogonal conjugation-assisted purification workflow [37]. Detected proteins without these pieces of information can be ambiguously identified as CSPs.

In the present study, to overcome the above-mentioned methodological and interpretation disadvantages, we focus on the identification of the Sulfo-NHS-SS-biotin labeled peptides from the CSPs. Steps of the presented high-throughput method were optimized to identify as many labeled/biotinylated peptides as possible from the cell surface in a single HPLC-MS/MS run.

The optimized method allows the characterization of the CSPs and the identification of several hundreds of extracellular regions belonging to various transmembrane proteins (TMPs, the most important subclass of the CSPs that have at least one transmembrane segment). Although their high-resolution structural determinations are constantly evolving by emerging new biotechnological and artificial intelligence tools such as Cryo-EM and natural language models utilized by AlphaFold2 [38], other bioinformatical [39] and experimental methods [10,40] still have a great role in topology characterization of TMPs (topology defines the number of transmembrane segments and the orientation of the connecting loop relative to the membrane). While most of the existing experimental approaches often produce few topology data for only one TMP, the work presented here can produce several data for dozens of proteins at the same time.

To the best of our knowledge, this method is the first attempt to optimize the primary amino group-specific labeling from the sample preparation to the mass spectrometry analysis in order to produce more specific and comprehensive extracellular labeled regions/peptides for different CSPs from a particular cell type. According to the TMPs, these new experimental data can be used as extracellular constraints in the CCTOP algorithm [39] to achieve a more accurate prediction for them. Fine-tuning the method can reveal new accessible protein regions on the cell surface that can be useful for the specific antibody, peptide, small molecule, or even drug design.

## 2. Results

**Figure 1:**
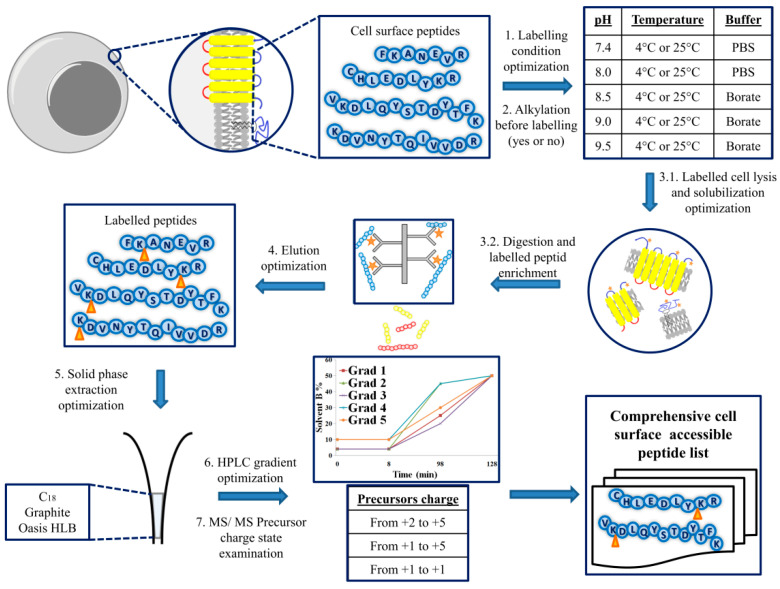
Flowchart of the optimized cell surface peptide characterization method. Cell surface proteins of the isolated cells are modified with a membrane-impermeable, primary amino group-specific biotinylation agent (Sulfo-NHS-SS-biotin) in different labeling conditions (the upper right corner). The efficiency of the cell surface peptide modification is monitored with or without alkylating agent (iodoacetamide) under the cell isolation process using the most efficient labeling condition. Thereafter, the cells are lysed, and plasma membranes are isolated and solubilized, avoiding endogen proteolysis under these steps. Then membrane-binding proteins (as transmembrane or other membrane-associated proteins) are digested with trypsin in a detergent-containing environment, and the modified peptides are purified on an optimal amount of high-capacity neutravidin agarose resin. Then biotinylated peptides are eluted by two consecutive incubations with DTT (dithiothreitol; the elution condition is optimized with biotinylated bovine serum albumin). Modified extracellular peptides are purified on one of the three different solid phases (performance of four methods investigated) that can be specific for different kinds of peptides. Finally, the enriched peptides are analyzed using nanoHPLC-MS/MS, examining various chromatographic separations (performance of five different gradients investigated) and adjusting the precursor charge state preferences (three setting parameters investigated) under tandem mass spectrometry analysis to find the optimal parameters of the developed method for the comprehensive modified peptide identification from the model cell surface. The light blue color marks the extracellular segments of the cell surface proteins. Furthermore, in the case of TMPs, yellows are transmembrane segments, and reds are their intracellular parts. The orange stars indicate the Sulfo-NHS-SS-biotin labeling, and the orange triangles after the elution are the remnant region from the labeling agent on the appropriate residues. The numbered parts around the blue arrows are the optimized steps in the method presented here.

We optimized several steps of the developed method to increase the number of labeled peptides identified from the cell surface in a single mass spectrometry run (these steps are numbered on the flowchart in Figure 1).

The next sections are divided according to the optimized steps and discuss in detail the control experiments used (dot blot, Western blot, SDS-PAGE, etc.) as well as their results. Finally, we summarize all the identified cell surface labeled peptides from CSPs for the model HL60 cell line and assess the membrane- and cell surface-protein specificity of the developed method. The correctness of extracellularly labeled positions from transmembrane proteins was checked using other experimental topology data collected in TOPDB [41].

### 2.1. Labeling Condition Optimization and the Effect of Alkylation for the Biotinylation

**Figure 2:**
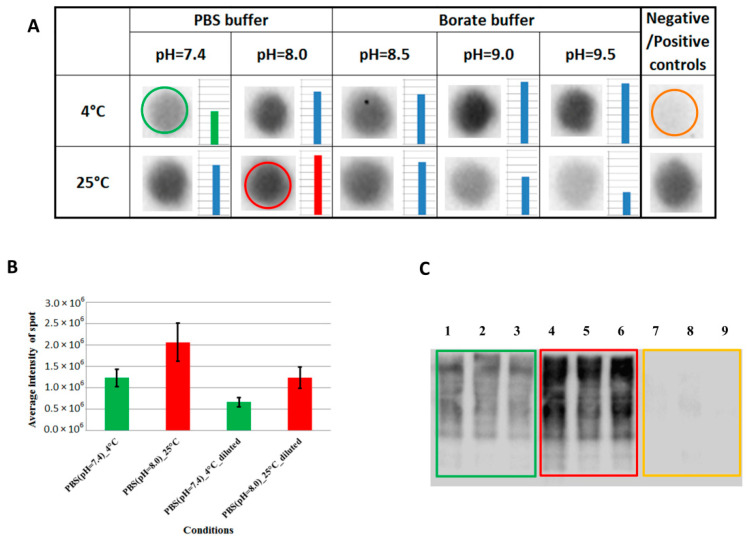
Dot blot and Western blot analysis of the biotinylation efficiency of the HL60 cell surface proteins. (**A**) HL60 cells were labeled in different buffer conditions at 4 °C or 25 °C during the same time intervals, and the biotin content of their membrane preparations was analyzed by dot blot (positive control: biotinylated BSA, negative control: non-biotinylated membrane preparations). The result of the “PBS (pH = 7.4)_4 °C” condition (which is the most commonly used in the cell surface labeling experiments) is indicated by a green circle, while the “PBS (pH = 8.0)_25 °C” condition is labeled by red (this was analyzed further in the present work), and the negative control is an orange circle. Intensities of the chemiluminescent signal were quantified by Image Lab 6.0 software and are presented to the right of the spots. The original dot blot is presented in Figure S10. (**B**) Dot blot analysis for a more accurate comparison of the two highlighted conditions. The original blotted PVDF membrane is in Figure S2 (where replicates from the appropriate conditions are similarly colored as above and every second spot (from left to right) is 60% of the previous one). The intensity of the spots was also analyzed by Image Lab 6.0 software and average chemiluminescent signals and their standard deviations are displayed on the chart (first two columns calculated from the first/more concentrated spots, “diluted” columns evaluated from every second spot, from left to right). (**C**) Western blot analysis to confirm the effectiveness of the labeling (colors indicate the type of the sample as above). 1–3: HL60 biotinylated (pH = 7.4; 4 °C) membrane preparations; 4–6: HL60 biotinylated (pH = 8.0; 25 °C) membrane preparations; 7–9: HL60 non-biotinylated membrane preparations (complementary SDS-PAGE without the negative control is in Figure S11, and the original Western blot is presented in Figure S14.). All the blots were captured by a Bio-Rad ChemiDoc XRS+ Imaging system.

Before extending the biotinylation method for labeling primary amino groups of CSPs on a larger number of living cells, it was essential to determine the efficiency of the modification using different conditions. Sulfo-NHS-SS-biotin reagent mainly targets lysines (prioritizing the primary amino groups of their side chains). Deprotonated primary amino groups in the extracellular regions are required for the reaction. Deprotonation can be facilitated by increasing the pH (Figure S1). Therefore, we applied five different pH conditions using two different buffers at two different temperatures (as indicated in Figure 2A) and then measured the biotinylation efficiency of the CSPs by dot blot or Western blot (as described in Supplementary Methods/Temperature and pH-dependence of the cell surface labeling). The biotinylated spots/lanes were visualized by HRP conjugated avidin and its chemiluminescent substrate (Figure 2), and the intensity of the chemiluminescent signal was quantified (Figure 2A,B).

We successfully modified the amino groups on the cell surface of the model HL60 cells under all tested experimental conditions (Figure 2A). The labeling efficiency was increased by raising the pH of the used buffers at 4 °C; at room temperature the highest efficiency was achieved at pH 8.0. To minimize the complexity of parameter optimization, we decided to use PBS buffer in the cell surface labeling process, but the pH value and the temperature were increased to 8.0 and 25 °C. To prove that the labeling efficiency can be much higher at pH = 8.0 and at 25 °C, we performed further dot blot and Western blot analyses (Figure 2C and Figure S2). The presented results confirmed that the biotinylation level of cell surface proteins was increased more than twice (compared with those experiments where pH = 7.4 and 4 °C were applied) by using the new condition (as can be seen in Figure 2B,C). Thus, it was also used for further experiments. Cell surface biotinylation of HL60 cells was also verified by confocal microscopy and flow cytometry measurement using the determined optimal labeling condition (Figures S12 and S13).

Next, we wanted to determine the effects of the alkylation step (in the last washing step) on the efficiency of the surface biotinylation. Thus, experiments were designed with or without alkylation (cells were incubated or not for 20 min with the wash buffer, details in the Supplementary Methods). These conditions were tested before the cell surface biotinylation, and the efficiency of the labeling was then also monitored by dot blot. The semi-quantitative results indicate the drawback of this alkylation step for the cell surface labeling and that the PBS washing for 20 min also decreases the efficiency of the biotinylation (it was more than two times lower in the case of the alkylation, Figure S3). Thus, these two steps were eliminated from the workflow in further experiments.

### 2.2. Optimization of Labeled Cell Lysis, Membrane Preparation Solubilization and Enrichment of Extracellular Protein Segments

In our earlier experiments we often found non-tryptic cleavage ends of peptides based on tandem mass spectrometry analysis. Therefore, we assumed the presence of endogenous proteases in membrane preparations after the cell lysis. To confirm this finding, we applied SDS-PAGE. Membrane preparations containing labeled and unlabeled proteins were loaded onto the gels from which two-two samples were pre-incubated for 1 h at 37 °C providing an ideal digestive condition. The protein bands were visualized on the gel utilizing Coomassie Brilliant Blue staining (Figure S4), and the results indicate that various endogenous proteases can be present in the samples. To avoid unpredictable effects, we optimized several steps of the labeled cell lysis and membrane preparation processes compared with our previous works [10,19] (details in the Methods Section). The labeled membrane preparations were solubilized, which was followed by proteolytic digestion using MS Grade trypsin. The new protocol resulted in between ~80 and 90% peptides with tryptic termini via the tandem mass spectrometry analysis (Figure S5), which confirms that a significant portion of the uncontrolled proteolytic cleavages were eliminated using the method described.

After digestion, the biotinylated peptides of the CSPs were enriched on a neutravidin agarose-containing column. The amount of resin was chosen to bind as many biotinylated components as possible. The biotin contents of the affinity column fractions before and after were monitored (Figure S6), and we did not find a detectable biotin signal in the case of the flow-through fractions for any of the samples (so all of the labeled peptides were bound on the column).

### 2.3. Cell Surface Peptide Elution Optimization and Their Solid-Phase Extraction Purification

Covalently biotinylated cell surface peptides were eluted by two consecutive DTT incubations of 60 min each. Elution conditions were set by using biotinylated BSA (details in Supplementary Methods/Labeling the primary amino groups of a model protein), and SDS-PAGE experiments were used to confirm the usefulness of the second elution (Figure S7). This was further confirmed by tandem mass spectrometry measurements. A total of 192 surface proteins with 1484 extracellularly modified peptides could be identified solely from the second elution fraction. These proteins were considered to have at least one labeled site (remnant motifs from the labeling agent after the reduction were either by ~88 Da/non-alkylated forms or ~145 Da/alkylated forms) in at least three separate MS runs (Table S4/Second elution sheet). A two-step elution was applied and the eluted fractions were combined in the later experiments.

**Figure 3:**
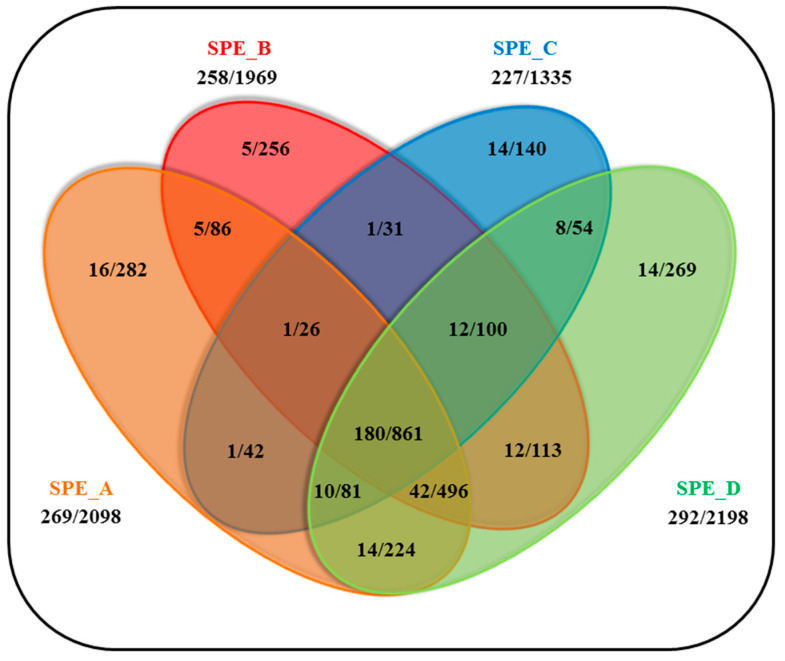
Venn diagram showing the number of individually labeled CSPs and the number of their labeled peptides in the various SPE experiments. Only those proteins (and their peptides) were taken into account that have been identified with at least one labeled position (i.e., positions that have been detected at least three times).

Peptides are usually enriched or desalted by various solid-phase extraction (SPE) strategies before HPLC-MS/MS analysis. We compared four different SPE methods using the above-mentioned peptide mixtures to identify the optimal one for the purification of labeled peptides from the HL60 cell surface. The four types of SPEs were marked with A, B, C and D and are described in detail in Table S1 (the marks are independent of the order presented there). Potential cell surface peptides are listed in Table S4/SPE sheet and depicted in a Venn diagram (Figure 3) showing the number of individually labeled CSPs and their extracellularly labeled peptides.

The SPE_D method was found to give the best results according to the number of the labeled proteins and peptides, although the other methods also provide unique labeled proteins and positions that can give additional information about the cell surface protein/peptide pool. However, the difference in selectivity is quite small (ca. 8% gain in the number of identified CSPs) and comparable to the variance of the DDA spectrum acquisition method. Therefore, no combination of these methods is advantageous; thus, SPE_D method was incorporated into the workflow.

### 2.4. HPLC Gradient Optimization and Precursor Charge Preference Setting under the HPLC-MS/MS Analysis

Sulfo-NHS-SS-biotin has been used for a long time in cell surface proteomics. However, the effects of these modifications on the chromatographic separation and ionization of the labeling residual motif-containing peptides have not been thoroughly studied yet. Here we examined five different chromatographic gradients (marked by Grad_A, B, C, D, and E) in separate experiments to find which is the most effective for labeled peptide identification. The results were similarly assessed (Table S5/Grad sheet, Figure S8) as in the SPE experiments and it was found that Grad A resulted in the most modified cell surface peptides per experiment.

Next, we analyzed the effects of three different precursor charge state preference settings for the MS/MS fragmentation. These ranges were from +1 to +5, only +1 or from +2 to +5 in the middle case focusing on the assumed singly charged precursors (the analysis identifiers are Charge_A, B, and C, the marks are independent of the order). No significant difference was found between the yields of the different analysis types but Charge_A resulted in slightly more labeled peptides (Table S5/Charge sheet, Figure S9).

### 2.5. Assessment of Specificity and Validation of the Developed Workflow

**Figure 4:**
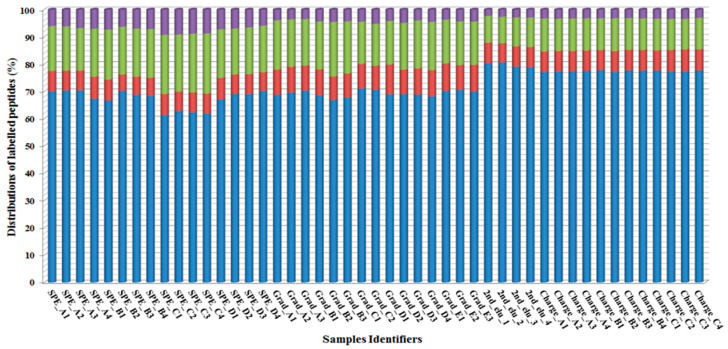
The distribution of cell surface labeled peptides per sample. The blue, red, green and purple color bars mark the percentage distributions of the labeled peptides from TMPs, non-TM_Subcellular_surface proteins, non-TM_GO_surface proteins and non-TM_non-surface proteins, respectively.

Altogether, more than half a million (redundant) peptides were sequenced in the 47 nanoHPLC-MS/MS runs (Tables S4 and S5), from which 191,334 peptides were labeled by Sulfo-NHS-SS-biotin from 557 individual proteins (Table S6). In the further evaluation, only those proteins were considered that had at least one extracellularly labeled position, and that position was identified at least three times. Applying this filter resulted in 1596 modified positions from 415 proteins. These proteins were classified into transmembrane or non-transmembrane protein groups by the CCTOP algorithm. Non-transmembrane proteins were divided into three further groups based on the UniProt annotation (as described in the Methods Section/Assessment of identified proteins and peptides) as subcellular localization is surface, GO annotation is surface and others (these are named in Figure 4 as non-TM_Subcellular_surface, non-TM_GO_surface and non-TM_non-surface, respectively; Table S6). Distributions of the labeled peptides per sample for these clustered proteins are depicted in Figure 4 to evaluate the membrane and surface specificity of the developed workflow.

The various mass spectrometry measurements resulted in ~60–80% biotinylated peptides from transmembrane proteins that confirm the membrane protein specificity of the presented method. According to the annotation of the labeled non-transmembrane peptides, most of them are cell surface proteins, which further strengthens the specificity of our method. We selected one of the most frequently captured proteins (based on Table S7), namely CD45 protein (Leucocyte common antigen), which was detected directly on the HL60 cell surface by the Alexa Fluor 488-conjugated CD45 antibody by confocal microscopy and by flow cytometry, as can be seen in the Figure S13A. These are reinforced by our tandem mass spectrometry runs since all of them resulted in labeled CD45 peptides.

To measure the topological accuracy of the developed method, we compared the location of labeled residues with the results of independent experiments that are listed in the TOPDB [41]. Only those labeled sites of TMPs that were detected at least three times in the nanoHPLC-MS/MS runs were considered (838 sites listed in Table S7). We excluded those proteins from the evaluation that could have originated from intracellular compartments (such as the endoplasmic reticulum, mitochondria, etc.; highlighted with grey background in the table) of damaged cells. Applying this filter resulted in 784 labeled positions. Previous experimental data confirm that these labeled positions are almost exclusively located in the extracellular regions (only six conflicting positions were found), proving our method almost 100% accurate and making it suitable for cell surface accessible peptide segment characterization (based on the ‘Topology verification’ column in Table S7). Using the TmAlphaFold database [42], the localizations of these labeled residues are visualized with cyan colored atoms on the predicted topology and 3D structure of the proteins, which are accessible via hyperlinks in the first and fourth columns in Table S7.

## 3. Discussion

In recent decades various experimental methods have been developed for the characterization of CSPs of different cell types or pathogens, as in the case of breast cancer cell lines [8], myogenic progenitors [43], *Listeria monocytogenes* [44], and methicillin-resistant *Staphylococcus aureus* [45]. Although the experimental procedures for their characterization are constantly evolving, the resulting protein lists of these methods are often contaminated by abundant cytoskeletal and other proteins and give limited information for the separation of valid hits from these proteins. Thus, the interpretation of the results is often controversial. Therefore, the development of more accurate and comprehensive methods remains vital for the characterization of the cell surface proteome of individual cells.

In the present work, we introduce a highly optimized high-throughput cell surface protein characterization method that is based on Sulfo-NHS-SS-biotin labeling of primary amino groups containing segments on the model HL60 cell surface. The essence of the developed method is that the extracellularly labeled proteins are easily separated from others that are ambiguously identified as CSPs. We focus on the identification of the extracellularly biotinylated protein segments and use them as an internal quality control. Thus, the individual steps of the method were fine-tuned to maximize the number of extracellularly localized and labeled peptides.

The membrane impermeable Sulfo-NHS-SS-biotin reagent has a N-hydroxysuccinimide (NHS) ester terminal that can react with deprotonated primary amino groups (the amine nucleophiles can attack at the electron-deficient carbonyl of the active ester). Therefore, the pH-dependent deprotonation of the primarily targeted lysine amino groups was analyzed by MarvinSketch software (ChemAxon, version 19.2.0, Figure S1). It was found that the increasing pH can enhance the deprotonation, and thereby, facilitate the efficiency of the cell surface labeling. Based on this observation and the fact that the NHS-Pegylation of a particular target protein is also pH-dependent [46], we hypothesized that the efficiency of the cell surface labeling with the mentioned chemical reagent is also pH-dependent, so first we analyzed various conditions in the labeling process. The results indicate that the labeling reaction was made more effective at both 4 °C and 25 °C by using increased pH in the appropriate buffer. Although in the latter case, at pH above 8.0, decreasing biotinylation was detected. This is presumably because the NHS-ester moieties of the labeling agent are hydrolysed more readily with increasing pH at this temperature, which competed with the biotinylation reaction (similar to the case of sulfo-NHS-LC-biotin [47]). Considering that we worked with living cells in isotonic solution and that we wanted to minimize the damage to cell integrity, we chose slightly alkaline (pH = 8.0) conditions at room temperature for the further labeling experiments. This resulted in near maximal labeling efficiency compared with the other examined conditions. Cell surface labeling was confirmed by confocal microscopy and flow cytometry (Figure S12).

Iodoacetamide is an often used alkylating agent which prevents any further disulfide bond rearrangement [48], so we used it in the last washing step of cell isolation before the Sulfo-NHS-SS-biotin incubation to ensure the protection of the disulfide-containing labeling agent. The optimized labeling was tested with or without alkylation agent under the cell isolation process to identify the effect of the iodoacetamide reagent on the primary amino group modification. The results of the experiments indicated that cell surface biotinylation is more effective without alkylation, and thus, it was excluded in the further sample preparation process. Although the exact cause of this finding has not been identified yet, side reactions may occur at the targeted lysine or peptide/protein-N-termini [49], which can prevent the biotinylation reaction.

Based on our previous experiments [10,19], cell surface peptides are often detected by semi-specific termini (not only lysine or arginine cleavage sites were identified by mass spectrometry after trypsin digestion). Thus we aimed to reduce them. The presence of the endogenous proteases was verified in the membrane preparations. Therefore the cell lysis, and membrane preparation steps of the method were carefully improved to avoid their unexpected digestion. The new protocol resulted in between ~85 and 90% peptides with fully specific termini (based on ~half a million identified peptides in 47 MS/MS runs). This new protocol paves the way for utilizing more specific proteases that are often used in bottom-up mass spectrometry measurement [50].

Biotinylated peptides were enriched on an affinity column, and we also determined the amount of high-capacity neutravidin agarose required to bind the total biotin content of each digested protein mixture (Figure S6). Because various elution parameters using reversible biotinylation have been described in the literature [8,19,51], the optimal elution was also set by using a model biotinylated BSA protein. After two consecutive incubations with 10 mM DTT, almost all the biotinylated components were removed from the affinity column.

We compared four different SPE techniques to clean-up and pre-concentrate the peptides before tandem mass spectrometry, investigating their effect on the amount of detected labeled peptides. All the tested methods proved to be applicable for the purification of labeled cell surface peptides, and most of the target compounds were identified using all the methods with minor differences regarding selectivity. However, C_18_-based methods showed superiority to graphite-based and HLB methods.

The amino group labeling has a disadvantage for tryptic proteolysis as it results in missed cleavages due to the modified moieties. These lead to longer and more complex peptides, making the MS/MS sequencing and identification more difficult or even impossible [10]. Additionally, the labeling may decrease the charge state of the peptide precursors because it modifies the amino group side chain of lysine residues. It has been observed that the detectability of similar biotin tags containing peptides can be promoted by chromatography gradient optimization and/or by taking into account singly-charged precursors in the tandem mass spectrometry analysis [52]. We hypothesize that these factors may affect the yields of the labeled peptides/proteins from our approach. Regarding this, five different gradients and three different ranges of precursor charge preference were analyzed to find the highest yield. Grad_A and Charge_A were found to be the best based on Figures S8 and S9. In our experience, including singly-charged precursors in the analysis did not provide a gain in efficiency, contrary to what was shown in a recent publication [52]. However, gradient slopes had a remarkable effect; the maximized distribution of peaks in the hydrophilic (lower retention times) and the hydrophobic (higher retention times) regions reached using Grad_A resulted in a moderately large increase in the number of identified TM proteins.

According to all the produced data, the developed method identified several hundred cell surface labeled proteins that have at least one labeled site on the surface of HL60 cells. CD45 is one of the most labeled proteins in the present work and it was further analyzed by confocal microscopy and flow cytometry using a specific antibody. These analyses proved the surface localization of this protein in HL60 cells (Figure S13). Transmembrane proteins were identified by the CCTOP algorithm, whereas for non-TM proteins, UniProt annotations were utilized. Regarding these analyses, it was clearly shown that the developed method has strong specificity for membrane and surface-anchored proteins (Figure 4). Besides the extracellular part of the CSPs, some abundant intracellular proteins were also labeled. These mainly originate from neutrophil extracellular traps that are specific for neutrophils [53] or differentiated HL60 cells [54] containing DNA, histones and cell-specific granule proteins [55].

Most of the labeled peptides belong to TMPs. To measure the accuracy of the presented protocol, the localization of labeled TM peptides was further analyzed. Out of 784 positions, 770 were characterized previously in the TOPDB database, and 99.2% of them were previously located in the extracellular region. These independent experiments validate the protocol presented here and show that the developed method is highly accurate for labeling the extracellular region of CSPs.

We would like to highlight that the new protocol is highly optimized and more robust than protocols of similar studies [19,20], as described in Table S3. The method can become a widely used tool for the comprehensive characterization of suitable cell surfaces, complementing and/or strengthening existing knowledge.

## 4. Materials and Methods

### 4.1. Cell Culture

The HL60 cells used in each experiment were obtained from the American Cell Type Culture Collection (ATCC CCL-240, Manassas, VA, USA). The cells were grown in Roswell Park Memorial Institute (RPMI) medium (Gibco, RPMI 1640 Medium, GlutaMAX Supplement, HEPES, Thermo Fisher Scientific (Waltham, MA, USA)) containing 10% fetal bovine serum (FBS, Gibco, Thermo Fisher Scientific) and 50 µg/mL of penicillin-streptomycin (Gibco, Thermo Fisher Scientific). The cells were maintained in a humidified incubator with 5% CO_2_ at 37 °C (Eppendorf, Galaxy 170R). Filtered FBS was added in all freshly prepared media using a Millex-GP syringe filter unit (0.22 µm pore size, polyethersulfone membrane, Merck Millipore Ltd., Burlington, MA, USA) and 50 mL syringe (Henke-Ject Luer-lock, Tuttlingen, Germany). The HL60 cell populations were passaged every 2–3 days under a laminar box (ESCO Class II Bsc) and tested for mycoplasma contamination before each labeling experiment. Approximately 2–3 × 10^7^ HL60 cells were isolated and biotinylated for each membrane preparation, and 1–2 × 10^6^ cells were aliquoted per sample when the efficiency of cell surface labeling was examined via various factors.

### 4.2. Cell Isolation

The HL60 cells were isolated by methods similar to those described in our previous works [10,19]. The medium was discarded by centrifugation at 300× *g* for 3 min at 4  °C (Eppendorf centrifuge 5804 R, A-4-44 swing-bucket rotor). The resulting cell pellet was gently washed with ice-cold phosphate-buffered saline (4 °C, PBS; 137 mM NaCl, 2.7 mM KCl, 10 mM Na_2_HPO_4_ and 1.8 mM KH_2_PO_4_; pH = 7.4, ingredients were obtained from Sigma-Aldrich, St. Louis, MO, USA), and then the solution was spin down again. The volume of wash buffer was set depending on cell number (1 × 10^7^ cells/3 mL PBS), and this washing step was repeated twice. In some experiments, an alkylation agent was used in the last PBS solution (4 mM final concentration of iodoacetamide, Sigma-Aldrich) and the cells were incubated for 20 min at 4 °C in dark conditions (see Section 3).

### 4.3. Biotinylation of Accessible Free Amino Groups on the Surface of HL60 Cells

The membrane-impermeable Sulfo-NHS-SS-biotin (Thermo Fisher Scientific) agent was used to label the accessible primary amino groups on the cell surface. After the last wash step, iodoacetamide-treated or not treated HL60 cells were biotinylated with this chemical reagent. The labeling reaction was performed as described in our original works [10,19] with slight modifications because the labeling efficiency of the cell surface was optimized as described in the Supplementary Methods. In the present work, the cells were labeled with ~2 mM Sulfo-NHS-SS-biotin in PBS (pH was adjusted to 8.0 with NaOH (Sigma-Aldrich)) at room temperature with constant rotation for 20 min. Thereafter, 25 mM Tris buffered saline (10^7^ cells/2 mL TBS, 25 mM Tris, 150 mM NaCl; pH = 7.4, Thermo Fisher Scientific) was used to stop the labeling process. The biotinylated live cells were separated by low-speed centrifugation (300× *g* for 3 min at 4 °C) and washed again twice by TBS (10^7^ cells/3 mL TBS) to remove the excess of the labeling agent and the small number of damaged cells before cell lysis. The cell viability was monitored by trypan blue dye staining (Gibco, Thermo Fisher Scientific) after the labeling process.

### 4.4. Mechanical Cell Lysis and Membrane Preparation

The labeled HL60 cells were incubated in ice-cold hypotonic lysis buffer (20 mM Tris-HCl, 10 mM KCl, 20 mM sucrose; pH = 7.4, all components were obtained from Sigma-Aldrich) for 10 min at 4 °C in the presence of 10.4 mM iodoacetamide. Mechanical disruption of the HL60 cells was performed on ice, and there were then passed through a 26-gauge, 0.5-inch needle (BBraun) with a 5 mL syringe (at least 10 times). The intact cells, cell debris and nuclei were pelleted at 1700× *g* for 7 min at 4 °C. Subsequently, 90% of the supernatant was transferred to a 10.4 mL polycarbonate tube (Beckman Coulter, Miami, FL, USA), and the pelleted fractions were resuspended in the 10% portion of the remaining supernatant and transferred into a 1.5 mL Eppendorf tube. These solutions were ground manually using a plastic micro pestle (180° rotation in both directions, 40 times, Sigma-Aldrich) then passed through a needle as described above, and again centrifuged at 1700× *g* for 7 min at 4 °C. The supernatants were combined in a polycarbonate tube and centrifuged at 100,000× *g* for 1 h at 4 °C using a 70.1 Ti fixed rotor (Beckman Coulter) in an L7-55 ultracentrifuge (Beckman Coulter). The supernatant was discarded and the pellet was washed once with 10-times diluted lysis buffer without iodoacetamide (pH set to 7.7 by 1.7 M Tris stock solution). Finally, it was centrifuged again at 100,000× *g* for 1 h at 4 °C. The pellet was resuspended in the diluted lysis buffer and homogenized by 25 strokes with a Potter-Elvehjem PTFE pestle in a glass tube (2 mL, Sigma-Aldrich) on ice. The protein concentration of the preparations was measured by the method of Lowry et al. [56] (using bovine serum albumin as a standard, Sigma-Aldrich). Aliquots (with 100–200 µg protein content) were stored in a freezer set to maintain −80 °C until later analysis. The similarity of membrane preparations was monitored by SDS-PAGE (see Supplementary Methods).

### 4.5. Solubilization of Membrane Preparations and Digestion of Membrane-Associated Proteins

We applied the same protocol as previously [10,19] with slight modifications. Membrane preparations with a total protein amount of ~50–100 µg (determined by the Lowry method) were pre-processed for each mass spectrometry analysis. First, membrane preps in slightly alkaline lysis buffer were supplemented with 50 mM of ammonium-bicarbonate (NH_4_HCO_3_, pH = 8.0, Sigma-Aldrich). RapiGest anionic surfactant (Waters Corporation, Milford, MA, USA) at a final concentration of 0.1% (*w/v*) was used for sample solubilization in the presence of 1.25 mM iodoacetamide and 1.25 mM BHES (Sigma-Aldrich). The suspensions were sonicated for 5 × 1 min in an ultrasonic bath (ElmaSchmidbauer GmbH, Singen, Germany) at 4 °C, and then the samples were incubated on ice for 25 min (vortexing every 5 min for 10 s). The solubilized membrane proteins were denatured by heating for 5 min at 95 °C in a dry-block thermostat (Biosan, Riga, Latvia) and then incubated on ice for cooling. Thereafter, the samples were digested overnight (~16 h) at 37 °C. Proteomics grade trypsin (Sigma-Aldrich) was used in a 1:50 (*w/w*) enzyme-to-protein ratio. Digestion was stopped by heat inactivation for 10 min at 95 °C and by irreversible trypsin inhibitor (TLCK hydrochloride, ~50 µM final concentration, Sigma-Aldrich) for 45 min at 37 °C. The mixture was spun down at 13,400 rpm for 5 min in a benchtop centrifuge (MiniSpin, Eppendorf). The supernatant was then treated with PNGaseF (250 unit/sample, New England Biolabs, Ipswich, MA, USA) for 2 h at 37 °C. The digestions of the samples were examined by SDS-PAGE (see Supplementary Methods).

### 4.6. Enrichment of the Biotinylated Protein Segments on Neutravidin Agarose Resin

Sulfo-NHS-SS-biotin modified peptides were immobilized on a neutravidin agarose resin (the agarose resin was packed into a spin-column, Thermo Fisher Scientific). The optimal amount of neutravidin agarose resin was determined by dot-blot analysis (Supplementary Methods). The labeled samples were incubated on affinity columns at room temperature with gentle rotation for 1 h. The affinity columns were washed extensively to efficiently wash away non-labeled components. Three different wash buffers were used with at least 20 column volumes: 50 mM NH_4_HCO_3_, 1 M NaCl, and 100 mM NaHCO_3_ (obtained from Sigma-Aldrich). The biotinylated peptides were eluted by a reducing agent (10 mM Dithiothreitol (DTT), Thermo Fisher Scientific) in 50 mM NH_4_HCO_3_ buffer using two consecutive incubations of 60 min, each at 37 °C. These were followed by an alkylation step with 25 mM iodoacetamide in dark conditions at 37 °C for 45 min. The significance of the two consecutive elutions was verified by biotinylated bovine serum albumin (Supplementary Methods). The alkylated mixture was transferred to an ultrafiltration device (Microcron-10, nominal mass cutoff 10 kDa, Merck Millipore Ltd., Tullagreen, Carrigtwohill, Ireland) and centrifuged at 14,000× *g* for 20 min at 4 °C. The filter membrane was washed once by the addition of 100 µL 50 mM NH_4_HCO_3_. Finally, all the solutions that passed through the filter were combined and dried in a pre-heated vacuum concentrator (55 °C, Barnstead Genevac miVac, Ipswich, UK) and stored at −20 °C until further usage.

### 4.7. Peptide Purification by Solid-Phase Extraction

The cell surface captured peptide mixtures were purified by four different SPE methods (detailed protocols can be seen in Table S1) using three different SPE cartridges including a reversed-phase C_18_ spin column (Thermo Fisher Scientific), an Oasis hydrophilic-lipophilic balance (HLB) spin tip (Waters Corporation) and a mixed C_18_ + graphite spin tip (Glygen, Columbia, MD, USA).

### 4.8. Isolated Peptide Separation by Nanoflow Liquid Chromatography

The SPE purified peptides were dissolved in 30 µL injection solvent containing 98% H_2_O, 2% acetonitrile and 0.1% formic acid, and then 6 µL was added for the analysis. A Dionex UltiMate 3000 RSLCnano System (Sunnyvale, CA, USA) was used for peptide separation. The peptides were trapped on an Acclaim PepMap100 C_18_ Nano-Trap column (5 μm particle size, 100 Å pore size, 100 μm × 20 mm, Thermo Fisher Scientific) and separated using an Acquity UPLC M-Class Peptide BEH130 C_18_ column (1.7 μm particle size, 130 Å pore size, 75 μm × 25 cm, Waters). Five different gradients were applied in separate experiments (Solvent A: water + 0.1% (*v/v*) formic acid (FA); Solvent B: acetonitrile + 0.1% (*v/v*) FA). The B solvent content of the gradient elution is presented in Table S2. The solvent gradient was linear between two time points.

### 4.9. Peptide Identification by Tandem Mass Spectrometry

The nanoflow HPLC was coupled to the mass spectrometer with a CaptiveSpray nanoBooster ionization source (Bruker Daltonik GmbH, Bremen, Germany). The mass spectrometer was a Maxis II QTOF (Bruker Daltonik GmbH), and the data acquisition strategy was the data-dependent analysis (DDA). The spectra were collected using a fixed cycle time of 2.5 s (dynamic exclusion 2 min) and acquired at 3 Hz in the 150–2200 m/z mass range, while CID was performed at 4 or 16 Hz depending on the intensity of the precursor. The preferred charge states of precursors were mostly set from +1 to +5, but some samples were analyzed using only +1 or from +2 to +5.

The raw data were first recalibrated with Bruker Compass DataAnalysis software 4.3 (Bruker Daltonik GmbH). The peptides were identified by Byonic 4.2.10 software. The search engine parameters are detailed in Table S1.

### 4.10. Assessment of Identified Proteins and Peptides

The results were evaluated using a similar process to that used previously [10,19]. The peptides of the mass spectrometry runs were filtered for a |LogProb| value of at least 2, resulting in a false discovery rate ≤ 1%. The peptide lists from different measurements are presented in Tables S4 and S5 (as the second elution, various chromatography gradient, different precursor charge state preferences, and SPE measurements). The peptides carrying the artificial modifications by our labeling process (+87.998 Da or +145.020 Da) were filtered and listed in Table S6.

The protein list was filtered for those proteins that have at least one extracellularly modified position and were detected at least three times by this position in the mass spectrometry measurements. Transmembrane proteins were identified by the CCTOP algorithm [39], and non-transmembrane proteins were classified into three groups based on UniProt annotations: (1) non-TM_Subcellular_surface if the subcellular location of the UniProt entry contains the terms “Cell surface”, “Extracellular”, “Secreted” or “Cell membrane”; (2) non-TM_GO_surface if the gene ontology of the UniProt entry contains the terms “Extracellular” and “Cell surface”; and (3) non-TM_non-surface if the entry does not contain any of the previously listed terms.

The topological accuracy of labeled positions were evaluated based on earlier published experimental results collected in the TOPDB database [41]. These labeled positions of TMPs and their topological validation (‘Extracellular’: the position was extracellular in at least one former experiment; ‘Intracellular’: the position was intracellular in the previous experiments; ‘Unknown’: ‘no data available’) are listed in Table S7. Furthermore, in this table, each labeled position in the first column and the TmAlphaFold evaluation result in the fourth column are hyperlinked to the TmAlphaFold database [42], where the predicted topology and 3D structure of the labeled proteins can be found and the atoms of the labeled residues are shown with cyan balls.

## 5. Conclusions

Prior to the mass spectrometry analysis, we significantly decreased the complexity of the isolated cell surface peptide mixtures compared with previously published protocols that isolate whole proteins with the labeling agent. Instead of labeled CSPs, only labeled peptides were purified, resulting in a lower number of identified proteins but also ensuring their modified accessible segments on the model cell surface were found. Using the optimized method presented, we identified more than 1700 individual surface-associated labeled peptides (~6000–7000 redundant peptides) from the model cell surface in a single nanoHPLC-MS/MS run. This is unique and a nearly 20-times higher yield compared with our previous works. The advantage of the optimized Sulfo-NHS-SS-biotin labeling method is providing a reliable, highly selective CSP list using at least one extracellularly labeled position as a filter. In regards to TMPs, the method enables more accurate prediction of their topology using these modified peptide segments. The utilization of this optimized method for surface peptide characterization should greatly facilitate the identification by HPLC—MS/MS and could be useful to anyone working on molecular proteomics of various surfaces for discovering new potential markers.

## Data Availability

The raw data has been uploaded to the MassIVE data repository with the ID: MSV000090149.

## Supplementary Materials

The following supporting information can be downloaded at: https://www.mdpi.com/article/10.3390/ijms24010273/s1.

## References

[1] Székely V., Patik I., Ungvári O., Telbisz Á., Szakács G., Bakos É., Özvegy-Laczka C. (2020). Fluorescent Probes for the Dual Investigation of MRP2 and OATP1B1 Function and Drug Interactions. Eur. J. Pharm. Sci..

[2] Palm W., Thompson C.B. (2017). Nutrient Acquisition Strategies of Mammalian Cells. Nature.

[3] Chen S., Xu Z., Li S., Liang H., Zhang C., Wang Z., Li J., Li J., Yang H. (2022). Systematic Interrogation of Cellular Signaling in Live Cells Using a Membrane-Anchored DNA Multitasking Processor. Angew. Chem. Int. Ed. Engl..

[4] Barneh F., Jafari M., Mirzaie M. (2016). Updates on Drug-Target Network; Facilitating Polypharmacology and Data Integration by Growth of DrugBank Database. Brief. Bioinform..

[5] Bausch-Fluck D., Goldmann U., Müller S., van Oostrum M., Müller M., Schubert O.T., Wollscheid B. (2018). The in Silico Human Surfaceome. Proc. Natl. Acad. Sci. USA.

[6] Sau S., Alsaab H.O., Kashaw S.K., Tatiparti K., Iyer A.K. (2017). Advances in Antibody-Drug Conjugates: A New Era of Targeted Cancer Therapy. Drug Discov. Today.

[7] Martinez-Martin N. (2017). Technologies for Proteome-Wide Discovery of Extracellular Host-Pathogen Interactions. J. Immunol. Res..

[8] Li Y., Wang Y., Mao J., Yao Y., Wang K., Qiao Q., Fang Z., Ye M. (2019). Sensitive Profiling of Cell Surface Proteome by Using an Optimized Biotinylation Method. J. Proteom..

[9] Kuhlmann L., Cummins E., Samudio I., Kislinger T. (2018). Cell-Surface Proteomics for the Identification of Novel Therapeutic Targets in Cancer. Expert Rev. Proteom..

[10] Langó T., Pataki Z.G., Turiák L., Ács A., Varga J.K., Várady G., Kucsma N., Drahos L., Tusnády G.E. (2020). Partial Proteolysis Improves the Identification of the Extracellular Segments of Transmembrane Proteins by Surface Biotinylation. Sci. Rep..

[11] Bausch-Fluck D., Hofmann A., Wollscheid B. (2012). Cell Surface Capturing Technologies for the Surfaceome Discovery of Hepatocytes. Methods Mol. Biol..

[12] Li Y., Qin H., Ye M. (2020). An Overview on Enrichment Methods for Cell Surface Proteome Profiling. J. Sep. Sci..

[13] Geladaki A., Kočevar Britovšek N., Breckels L.M., Smith T.S., Vennard O.L., Mulvey C.M., Crook O.M., Gatto L., Lilley K.S. (2019). Combining LOPIT with Differential Ultracentrifugation for High-Resolution Spatial Proteomics. Nat. Commun..

[14] Wu C.C., MacCoss M.J., Howell K.E., Yates J.R. (2003). A Method for the Comprehensive Proteomic Analysis of Membrane Proteins. Nat. Biotechnol..

[15] Kim Y., Elschenbroich S., Sharma P., Sepiashvili L., Gramolini A.O., Kislinger T. (2011). Use of Colloidal Silica-Beads for the Isolation of Cell-Surface Proteins for Mass Spectrometry-Based Proteomics. Methods Mol. Biol..

[16] Wollscheid B., Bausch-Fluck D., Henderson C., O’Brien R., Bibel M., Schiess R., Aebersold R., Watts J.D. (2009). Mass-Spectrometric Identification and Relative Quantification of N-Linked Cell Surface Glycoproteins. Nat. Biotechnol..

[17] Zeng Y., Ramya T.N.C., Dirksen A., Dawson P.E., Paulson J.C. (2009). High-Efficiency Labeling of Sialylated Glycoproteins on Living Cells. Nat. Methods.

[18] Hörmann K., Stukalov A., Müller A.C., Heinz L.X., Superti-Furga G., Colinge J., Bennett K.L. (2016). A Surface Biotinylation Strategy for Reproducible Plasma Membrane Protein Purification and Tracking of Genetic and Drug-Induced Alterations. J. Proteome Res..

[19] Langó T., Róna G., Hunyadi-Gulyás É., Turiák L., Varga J., Dobson L., Várady G., Drahos L., Vértessy B.G., Medzihradszky K.F. (2017). Identification of Extracellular Segments by Mass Spectrometry Improves Topology Prediction of Transmembrane Proteins. Sci. Rep..

[20] Bausch-Fluck D., Hofmann A., Bock T., Frei A.P., Cerciello F., Jacobs A., Moest H., Omasits U., Gundry R.L., Yoon C. (2015). A Mass Spectrometric-Derived Cell Surface Protein Atlas. PLoS ONE.

[21] Cogger K.F., Sinha A., Sarangi F., McGaugh E.C., Saunders D., Dorrell C., Mejia-Guerrero S., Aghazadeh Y., Rourke J.L., Screaton R.A. (2017). Glycoprotein 2 Is a Specific Cell Surface Marker of Human Pancreatic Progenitors. Nat. Commun..

[22] Müller A., Langó T., Turiák L., Ács A., Várady G., Kucsma N., Drahos L., Tusnády G.E. (2019). Covalently Modified Carboxyl Side Chains on Cell Surface Leads to a Novel Method toward Topology Analysis of Transmembrane Proteins. Sci. Rep..

[23] Nagano K., Shinkawa T., Kato K., Inomata N., Yabuki N., Haramura M. (2011). Distinct Cell Surface Proteome Profiling by Biotin Labeling and Glycoprotein Capturing. J. Proteom..

[24] Boheler K.R., Gundry R.L. (2017). Concise Review: Cell Surface N-Linked Glycoproteins as Potential Stem Cell Markers and Drug Targets. Stem Cells Transl. Med..

[25] Goshe M.B., Blonder J., Smith R.D. (2003). Affinity Labeling of Highly Hydrophobic Integral Membrane Proteins for Proteome-Wide Analysis. J. Proteome Res..

[26] Roesli C., Mumprecht V., Neri D., Detmar M. (2008). Identification of the Surface-Accessible, Lineage-Specific Vascular Proteome by Two-Dimensional Peptide Mapping. FASEB J..

[27] Özkan Küçük N.E., Şanal E., Tan E., Mitchison T., Özlü N. (2018). Labeling Carboxyl Groups of Surface-Exposed Proteins Provides an Orthogonal Approach for Cell Surface Isolation. J. Proteome Res..

[28] DeChancie J., Houk K.N. (2007). The Origins of Femtomolar Protein-Ligand Binding: Hydrogen-Bond Cooperativity and Desolvation Energetics in the Biotin-(Strept)Avidin Binding Site. J. Am. Chem. Soc..

[29] Rösli C., Rybak J.-N., Neri D., Elia G. (2008). Quantitative Recovery of Biotinylated Proteins from Streptavidin-Based Affinity Chromatography Resins. Methods Mol. Biol..

[30] Rafiee M.-R., Sigismondo G., Kalxdorf M., Förster L., Brügger B., Béthune J., Krijgsveld J. (2020). Protease-Resistant Streptavidin for Interaction Proteomics. Mol. Syst. Biol..

[31] Karhemo P.-R., Ravela S., Laakso M., Ritamo I., Tatti O., Mäkinen S., Goodison S., Stenman U.-H., Hölttä E., Hautaniemi S. (2012). An Optimized Isolation of Biotinylated Cell Surface Proteins Reveals Novel Players in Cancer Metastasis. J. Proteom..

[32] Almahariq M., Chao C., Mei F.C., Hellmich M.R., Patrikeev I., Motamedi M., Cheng X. (2015). Pharmacological Inhibition and Genetic Knockdown of Exchange Protein Directly Activated by CAMP 1 Reduce Pancreatic Cancer Metastasis in Vivo. Mol. Pharmacol..

[33] Ravenhill B.J., Kanjee U., Ahouidi A., Nobre L., Williamson J., Goldberg J.M., Antrobus R., Dieye T., Duraisingh M.T., Weekes M.P. (2019). Quantitative Comparative Analysis of Human Erythrocyte Surface Proteins between Individuals from Two Genetically Distinct Populations. Commun. Biol..

[34] Walton A., Stes E., Cybulski N., Van Bel M., Iñigo S., Durand A.N., Timmerman E., Heyman J., Pauwels L., De Veylder L. (2016). It’s Time for Some “Site”-Seeing: Novel Tools to Monitor the Ubiquitin Landscape in Arabidopsis Thaliana. Plant Cell.

[35] Niehage C., Karbanová J., Steenblock C., Corbeil D., Hoflack B. (2016). Cell Surface Proteome of Dental Pulp Stem Cells Identified by Label-Free Mass Spectrometry. PLoS ONE.

[36] Smolders K., Lombaert N., Valkenborg D., Baggerman G., Arckens L. (2015). An Effective Plasma Membrane Proteomics Approach for Small Tissue Samples. Sci. Rep..

[37] Liu G., Choi M.H., Ma H., Guo X., Lo P.-C., Kim J., Zhang L. (2022). Bioorthogonal Conjugation-Assisted Purification Method for Profiling Cell Surface Proteome. Anal. Chem..

[38] Varadi M., Anyango S., Deshpande M., Nair S., Natassia C., Yordanova G., Yuan D., Stroe O., Wood G., Laydon A. (2022). AlphaFold Protein Structure Database: Massively Expanding the Structural Coverage of Protein-Sequence Space with High-Accuracy Models. Nucleic Acids Res..

[39] Dobson L., Reményi I., Tusnády G.E. (2015). CCTOP: A Consensus Constrained TOPology Prediction Web Server. Nucleic Acids Res..

[40] Schäfer K., Engstler C., Dischinger K., Carrie C. (2022). Assessment of Mitochondrial Protein Topology and Membrane Insertion. Methods Mol. Biol..

[41] Dobson L., Langó T., Reményi I., Tusnády G.E. (2015). Expediting Topology Data Gathering for the TOPDB Database. Nucleic Acids Res..

[42] Dobson L., Szekeres L.I., Gerdán C., Langó T., Zeke A., Tusnády G.E. (2022). TmAlphaFold Database: Membrane Localization and Evaluation of AlphaFold2 Predicted Alpha-Helical Transmembrane Protein Structures. Nucleic Acids Res..

[43] Tey S.-R., Mueller M., Reilly M., Switalski C., Robertson S., Sakanaka-Yokoyama M., Suzuki M. (2022). Cell Surface Proteins for Enrichment and In Vitro Characterization of Human Pluripotent Stem Cell-Derived Myogenic Progenitors. Stem Cells Int..

[44] Esbelin J., Santos T., Ribière C., Desvaux M., Viala D., Chambon C., Hébraud M. (2018). Comparison of Three Methods for Cell Surface Proteome Extraction of Listeria Monocytogenes Biofilms. OMICS.

[45] Monteiro R., Hébraud M., Chafsey I., Chambon C., Viala D., Torres C., Poeta P., Igrejas G. (2015). Surfaceome and Exoproteome of a Clinical Sequence Type 398 Methicillin Resistant Staphylococcus Aureus Strain. Biochem. Biophys. Rep..

[46] Nojima Y., Iguchi K., Suzuki Y., Sato A. (2009). The PH-Dependent Formation of PEGylated Bovine Lactoferrin by Branched Polyethylene Glycol (PEG)-N-Hydroxysuccinimide (NHS) Active Esters. Biol. Pharm. Bull..

[47] Grumbach I.M., Veh R.W. (1991). Sulpho-N-Hydroxysuccinimide Activated Long Chain Biotin. A New Microtitre Plate Assay for the Determination of Its Stability at Different PH Values and Its Reaction Rate with Protein Bound Amino Groups. J. Immunol. Methods.

[48] Denoncin K., Vertommen D., Paek E., Collet J.-F. (2010). The Protein-Disulfide Isomerase DsbC Cooperates with SurA and DsbA in the Assembly of the Essential β-Barrel Protein LptD. J. Biol. Chem..

[49] Suttapitugsakul S., Xiao H., Smeekens J., Wu R. (2017). Evaluation and Optimization of Reduction and Alkylation Methods to Maximize Peptide Identification with MS-Based Proteomics. Mol. Biosyst..

[50] Miller R.M., Millikin R.J., Hoffmann C.V., Solntsev S.K., Sheynkman G.M., Shortreed M.R., Smith L.M. (2019). Improved Protein Inference from Multiple Protease Bottom-Up Mass Spectrometry Data. J. Proteome Res..

[51] Dwivedi-Agnihotri H., Srivastava A., Shukla A.K. (2020). Reversible Biotinylation of Purified Proteins for Measuring Protein-Protein Interactions. Methods Enzymol..

[52] Nierves L., Lange P.F. (2021). Detectability of Biotin Tags by LC-MS/MS. J. Proteome Res..

[53] Singh A., Verma S., Modak S.B., Chaturvedi M.M., Purohit J.S. (2022). Extra-Nuclear Histones: Origin, Significance and Perspectives. Mol. Cell. Biochem..

[54] Guo Y., Gao F., Wang Q., Wang K., Pan S., Pan Z., Xu S., Li L., Zhao D. (2021). Differentiation of HL-60 Cells in Serum-Free Hematopoietic Cell Media Enhances the Production of Neutrophil Extracellular Traps. Exp. Ther. Med..

[55] Semeraro F., Ammollo C.T., Morrissey J.H., Dale G.L., Friese P., Esmon N.L., Esmon C.T. (2011). Extracellular Histones Promote Thrombin Generation through Platelet-Dependent Mechanisms: Involvement of Platelet TLR2 and TLR4. Blood.

[56] Lowry O.H., Rosebrough N.J., Farr A.L., Randall R.J. (1951). Protein Measurement with the Folin Phenol Reagent. J. Biol. Chem..

