# Supplementary material for: Comprehensive Discovery of the Accessible Primary Amino Group-Containing Segments from Cell Surface Proteins by Fine-Tuning a High-Throughput Biotinylation Method
Source: Int J Mol Sci. 2022 Dec 23;24(1):273. doi: 10.3390/ijms24010273 (PMC9820203; doi:10.3390/ijms24010273)
Supplement: Supplementary file 1 [file ijms-24-00273-s001.zip › ijms-2085155-supplementary.pdf]

Supplementary materials for

Comprehensive discovery of the accessible primary amino group-containing segments from cell surface proteins by fine-tuning a high-throughput biotinylation method

Tamás Langó [1,*], Katalin Kuffa [1,2], Gábor Tóth [3], Lilla Turiák [3], László Drahos [3], and Gábor E Tusnády [1]

[1] Protein Bioinformatics Research Group, Institute of Enzymology, Research Centre for Natural Sciences, Magyar Tudósok krt 2, H-1117 Budapest, Hungary

[2] Doctoral School of Biology and Institute of Biology, ELTE Eötvös Loránd University, Pázmány P. stny. 1/C, H-1117 Budapest, Hungary

[3] MS Proteomics Research Group, Institute of Organic Chemistry, Research Centre for Natural Sciences, Magyar Tudósok krt 2, H-1117 Budapest, Hungary

* Correspondence: lango.tamas@ttk.hu

Supplementary Methods

Temperature and pH-dependence of the cell surface labeling

2*10[6] HL60 cells/sample were isolated and washed as described in Methods/Cell isolation. Cells were labeled by Sulfo-NHS-SS-biotin in two different buffers (PBS or 50 mM Sodium tetraborate buffer (STB, Sigma-Aldrich)) at five various pH values (PBS at pH 7.4-8.0, STB at pH 8.5-9-9.5) in separate experiments. The labeling process was performed both at 4°C and at room temperature for 20 min. The process was quenched and cells were lysed similarly as described in Methods. The labeling efficiency of the membrane preparations was detected by dot blot assay. These preparations were diluted 50-fold in NH_4HCO_3 buffer and labeled components were directly immobilized onto a 0.45 μm PVDF membrane using a Bio-Dot microfiltration apparatus (Bio-Rad Laboratories, Hercules, Ca, USA). The PVDF membrane was blocked with 5% (m/V) BSA diluted in TBS-T buffer (25 mM Tris-HCl, pH=7.4, 2.7 mM KCl, 137 mM NaCl, 0.1% Tween-20 (Sigma-Aldrich)) for 12 hours at room temperature with constant agitation. Thereafter the membrane was washed by TBS-T three times (incubated for 10 min in each washing step), and treated by HRP conjugated avidin (diluted 50000-fold in the blocking buffer) for 45 min. For the visualization of the labeling efficiency the enhanced chemiluminescence reagent (two component: luminol and peroxide solution, Merck Millipore Ltd)

was applied. The images were captured by a ChemiDoc XRS+ Imaging system (Bio-Rad, Figure 2, Figure S2-S10).

Effect of iodoacetamide alkylation for cell surface labeling

1-1*10^6 HL60 cells were treated with or without 4 mM alkylation agent in the last washing step of the cell isolation and were incubated or not for 20 min in separate experiments. Labeling efficiency of the cell surface was monitored by dot blot as described above. The images were also captured by a ChemiDoc XRS+ Imaging system (Figure S3).

Proteins of the preparations and digestions analyzed by SDS-PAGE

Separating/resolving gels (12% Acrylamide/Bis (37,5/1, Bio-Rad) Tris buffer (375 mM Tris-HCl, pH=8.8), 0.1% (w/V) Sodium dodecyl sulfate (SDS; Sigma-Aldrich), 0.5% (w/w) ammonium persulfate (Sigma-Aldrich) and 0.05% (V/V) Tetramethylethylenediamine (Temed, Bio-Rad)) and stacking gels (similar composition to separating gel, except Tris buffer (125 mM, pH=6.8) and Acrylamide/Bis (4%)), were allowed to polymerize for 30 min at room temperature. Samples of the membrane preparations (with ~5-10 μg total protein content/well) were diluted in 4*sample buffer (containing 250 mM Tris base, 10% (w/V) SDS, 0.008% (m/V) Bromophenol blue (Bio-Rad) and 40% Glycerol (Thermo Fisher Scientific)), boiled for 5 min at 95°C in the presence or absence of DTT in a dry-block thermostat, finally loaded them to the wells. GRS Protein Marker Multicolour (GRiSP, Porto, Portugal) or Sharpmas VI protein MW marker (Euroclone, Milan, Italy) were used as molecular weight standards. Electrophoresis was performed for 10 min at 80 V, then for 60 min at 140 V in electrophoresis buffer (25 mM Tris base, 192 mM glycine (Sigma-Aldrich), 0.1% SDS pH= 8.7) using a PowerPac Universal power supply with Mini-Protean tetra vertical electrophoresis cell (Bio-Rad). After running, the gels were washed twice by ddH_2O and stained with Coomassie Brilliant Blue G-250 dye (Thermo Fisher Scientific) for 45 min. The gels were washed three times for 10 min by ddH_2O, and then separated proteins were detected by ChemiDoc XRS+ (Bio-Rad, Figure S4-S11).

Western blot analysis of biotinylated samples

After the protein separation was ended, the stacking region of the gel was cut off and the remained resolving gel was washed by ddH_2O until the front of the Bromophenol blue was disappeared. Gel was equilibrated in transfer buffer (25 mM Tris base, 192 mM glycine and 15% (V/V) methanol, pH=8.8) for 10 min, under the incubation sponges and filter papers were wet in transfer buffer and the PVDF membrane was wet in methanol. Then transfer sandwich (sequence was: sponge-3filter papers-gel-PVDF membrane-3filter papers-sponge) was created and blotted at ~70 V for 80 min on ice using

the PowerPac Universal power supply again. PVDF membrane was treated similarly as described in Supplementary Methods/ Temperature and pH dependence of the cell surface labeling. Biotinylated proteins were detected by HRP conjugated avidin, the images were captured by a ChemiDoc XRS+ Imaging system as above.

Labeling the primary amino groups of a model protein

Bovine Serum Albumin was applied as a model protein that was labeled or unlabeled in 3-3 independent replicates (250 µg BSA for each). BSA was incubated with or without 2 mM Sulfo-NHS-SS-biotin at room temperature for 20 min in PBS buffer (pH=8.0), the reaction was quenched by TBS buffer. Labeled or unlabeled BSA-containing solutions were transferred to Amicon Ultra-0.5 centrifugal filter units (nominal mass cutoff 10 kDa, Merck Millipore Ltd) and centrifuged at 14000 g for 10 min at 4°C. In order to wash away the excess labeling reagent, two additional TBS washing steps were used respectively, then three PBS washing steps were applied to reach the buffer exchange. Finally, biotinylated or unlabeled model proteins were recovered by reverse spin. Protein content was determined by Lowry method, biotinylation was analyzed by dot blot as described earlier. Labeled BSA was applied to monitoring the binding capacity of the affinity column and the success of the elution by DTT (Figure S7).

Biotinylated cell surface, CD45 protein and their co-localization were analyzed by confocal microscopy and by flow cytometry

Sulfo-NHS-SS-biotin labeling and CD45 surface localization were verified by confocal microscopy. Biotinylated cells were blocked with 0,5% (m/V) BSA in PBS for 30 minutes at room temperature then were incubated with FITC-conjugated anti-biotin antibody (Sigma-Aldrich, 1:100 for 30 min, at 37°C) or Allophycocyanin-conjugated streptavidin (Thermo Fisher Scientific, 1:100 for 30 min, at 37°C) in the CD45 co-localization experiment. CD45 protein were visualized by Alexa Fluor 488-conjugated CD45 antibody (Thermo Fisher Scientific, 2:100 for 30 min, at 37°C) after the cells were treated similarly as above. Images were acquired with a Zeiss LSCM 710 microscope using a 63x NA=1.4 Plan Apo objective (Figure S12A-S13). Flow cytometry measurements were carried out on these cells using the above mentioned FITC-conjugated anti-biotin antibody and A488-CD45 antibody (Figure S12B-S13A).

Supplementary Figures

Figure S1. The ionisation states of the lysine amino groups or carboxyl termini depending on pH

The presence of the deprotonated primary amino groups in the extracellular regions is essential to the reaction with NHS ester containing reagents (such as the Sulfo-NHS-SS-biotin labeling reagent in the present work) because the amine nucleophiles can attack at the electron-deficient carbonyl of the active ester. Therefore pH dependent ionization states of the targeted lysine residue were analyzed by MarvinSketch software (ChemAxon, version 19.2.0).

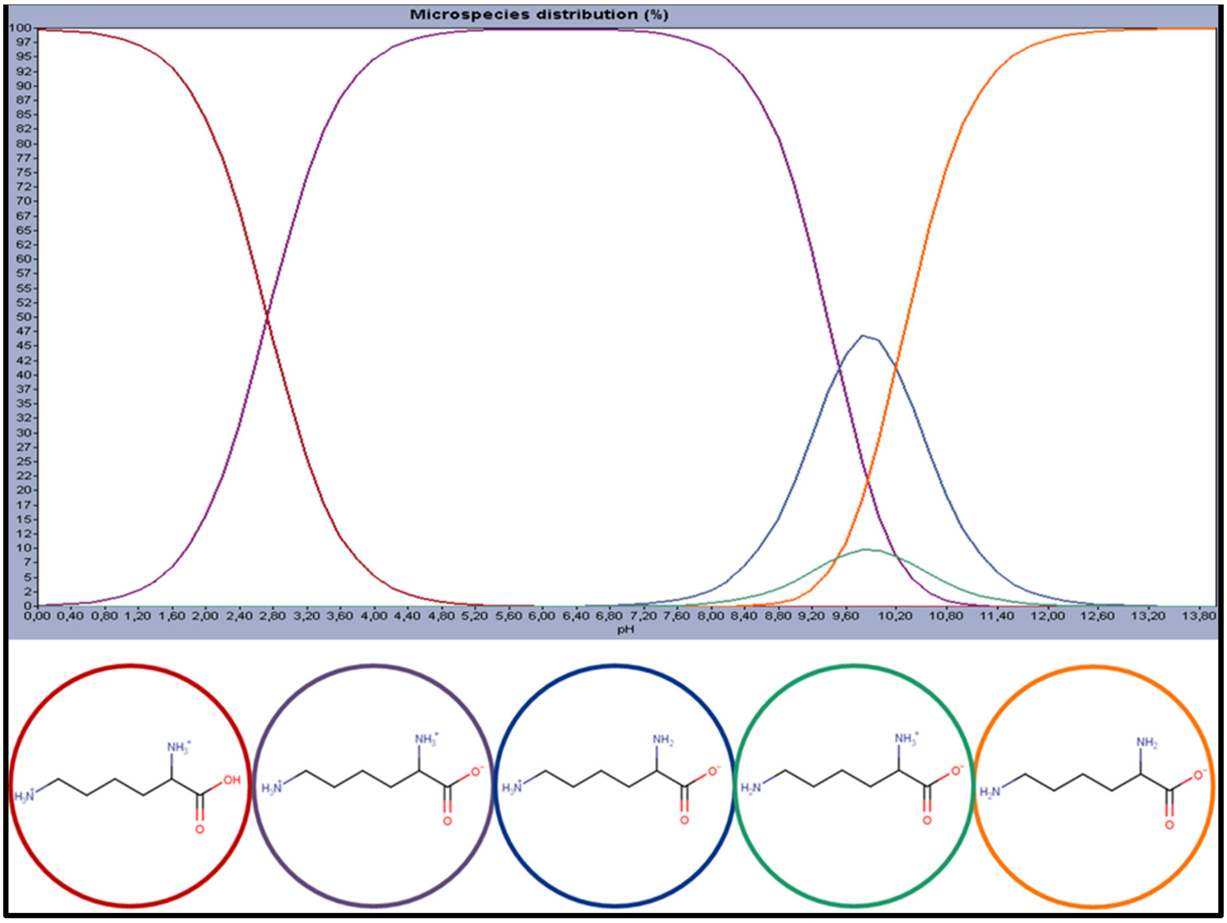

Figure S1. The ionic states of the lysine residues dependent on pH value of the buffer. The distribution of the lysine ionized states are indicted by different color lines: in the case of the red line, both amino groups are protonated and the carboxyl group is undissociated; the purple line both amino groups are protonated the carboxyl group is a carboxylate anion; the blue line the amino group of the side chain is protonated and the alpha carbon's amino group is deprotonated and carboxyl group is carboxylate; the green line the amino group of the side chain is deprotonated and the other is protonated, carboxyl group is carboxylate anion; the orange line both amino groups are deprotonated, the carboxyl group is in anionic form (pictures were captured from https://chemaxon.com/ by MarvinSketch 19.2.0 software)

Figure S2. Biotin contents of biotinylated membrane preparations were analyzed by dot blot using two different labeling conditions (based on Figure 2).

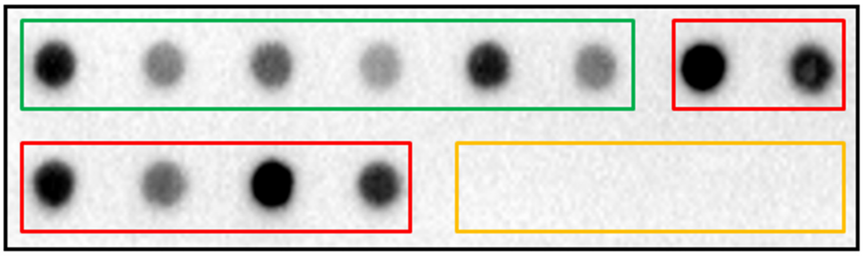

Figure S2. Dot blot analysis for a more accurate comparison of the biotinylation efficiency of the two highlighted conditions. Blotted PVDF membrane, replicates of the pH=7.4 at 4°C conditions are in green frame and replicates of the pH=8.0 at 25°C conditions are in the red frame and every second spot (from left to right) is 60% of the previous one), negative controls are in the orange frame.

Figure S3. Effect of iodoacetamide alkylation for cell surface labeling

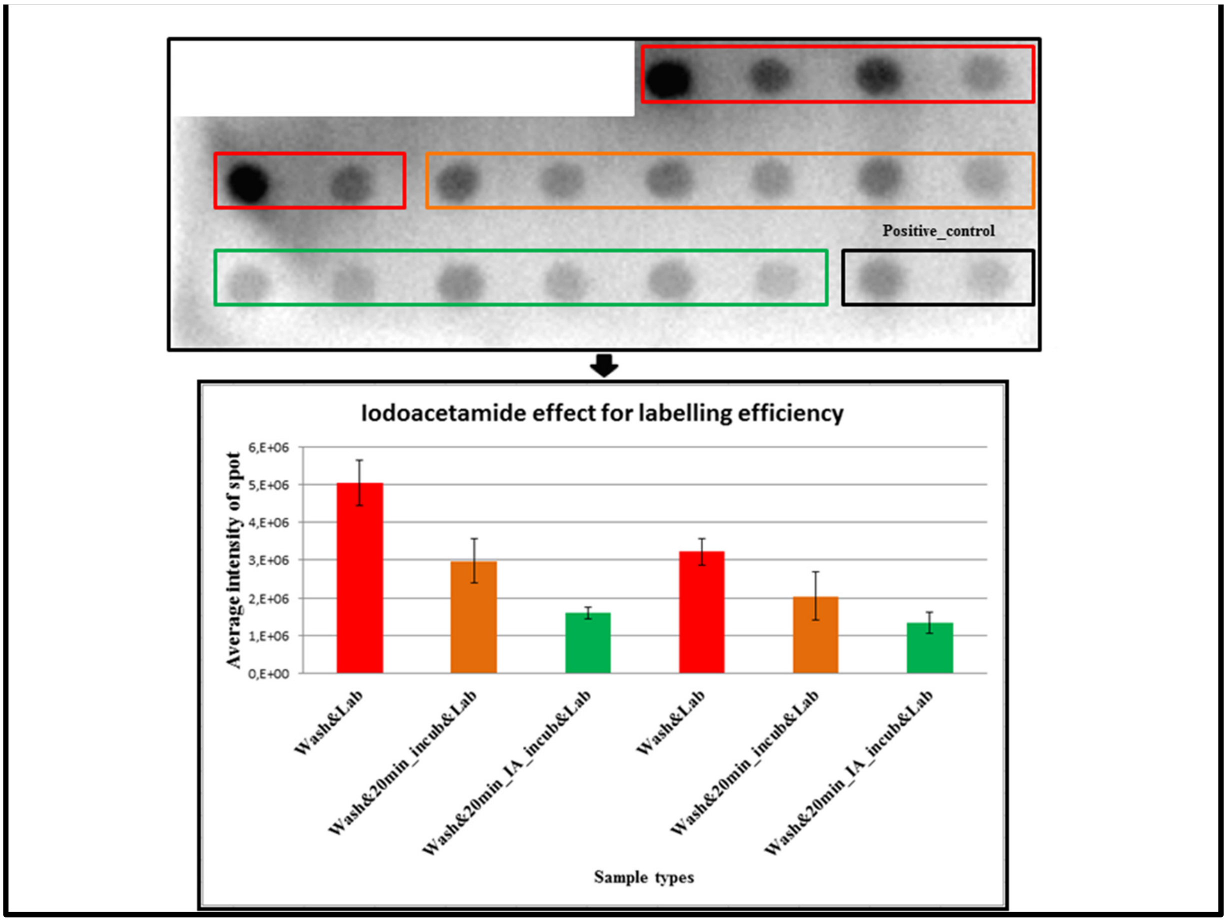

Figure S3. Dot blot analysis for the detection of the biotinylation efficiency on the surface of HL60 cells after the alkylation agent containing washing step. Three different conditions were used in the last washing process of cell isolation before the labeling as follows: cells were simply washed by PBS, incubated for 20 min in PBS and cells were alkylated by iodoacetamide in the last PBS washing for 20 min (they are colored red, orange and green, respectively). The dot blot results are presented in the upper part of the figure, in the colored, framed parts are seen the three-three biological replicates per sample types in two protein concentrations per replicates (from left to right, every second spots are the ¾ dilution of the previous one). The intensity of the spots were analyzed by Image Lab 6.0 software and average chemiluminescent signals and their standard deviations are displayed on the lower part (the first three columns calculated from the concentrated spots, the last three columns depicted from the diluted spots (every second spots in the frames; abbreviations on the diagram are as follows: IA: iodoacetamide, incub: incubation, Lab: labeled). The blot was captured by a Bio-Rad ChemiDoc XRS+ Imaging system.

Figure S4. SDS-PAGE was used to test the presence of endogenous proteases under the membrane preparation and solubilization processes

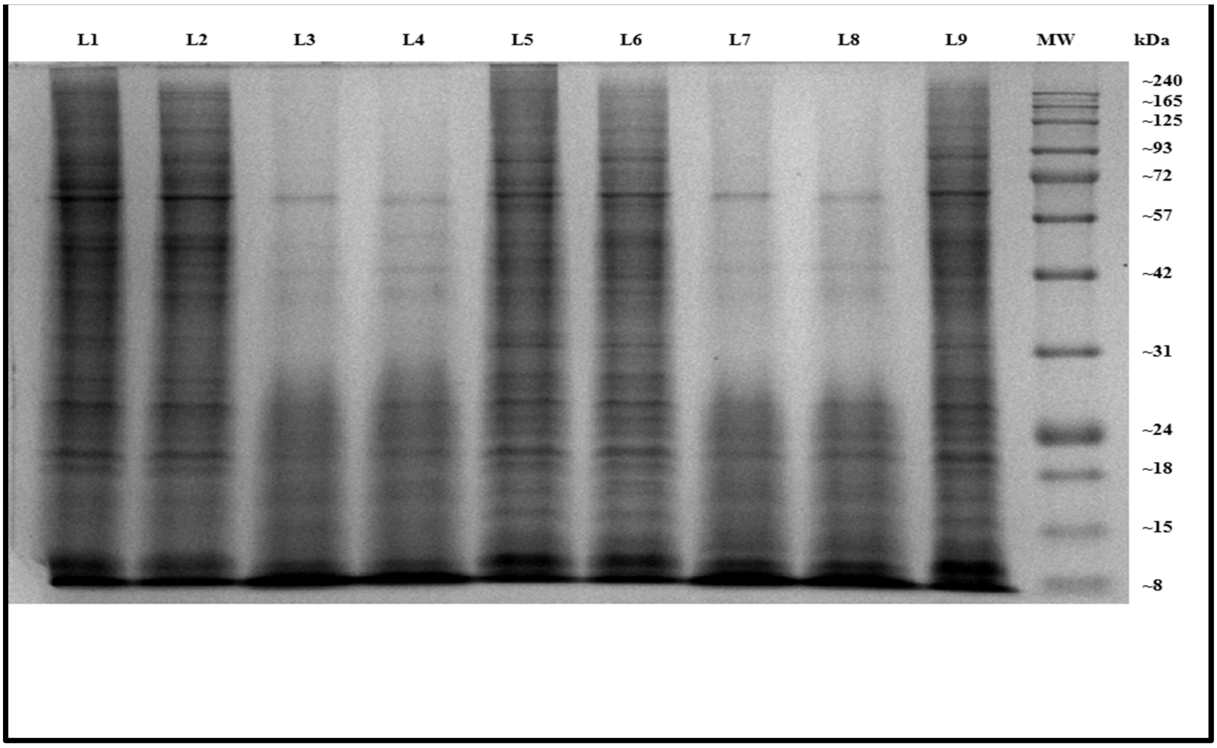

Figure S4. SDS-PAGE analysis to verify the presence of the endogenous proteases. Biotinylated (L5-L6-L7-L8-L9) and non-biotinylated (L1-L2-L3-L4) membrane preparations were solubilized and in some cases pre-incubated for one hour at 37°C (L3-L4-L7-L8). The protein pattern was analyzed by SDS-PAGE utilizing Coomassie Blue staining on the gel (MW: Sharpmas VI protein MW marker). The gel was captured by a Bio-Rad ChemiDoc XRS+ Imaging system.

Figure S5. Distributions of the terminal specificity of the identified peptides

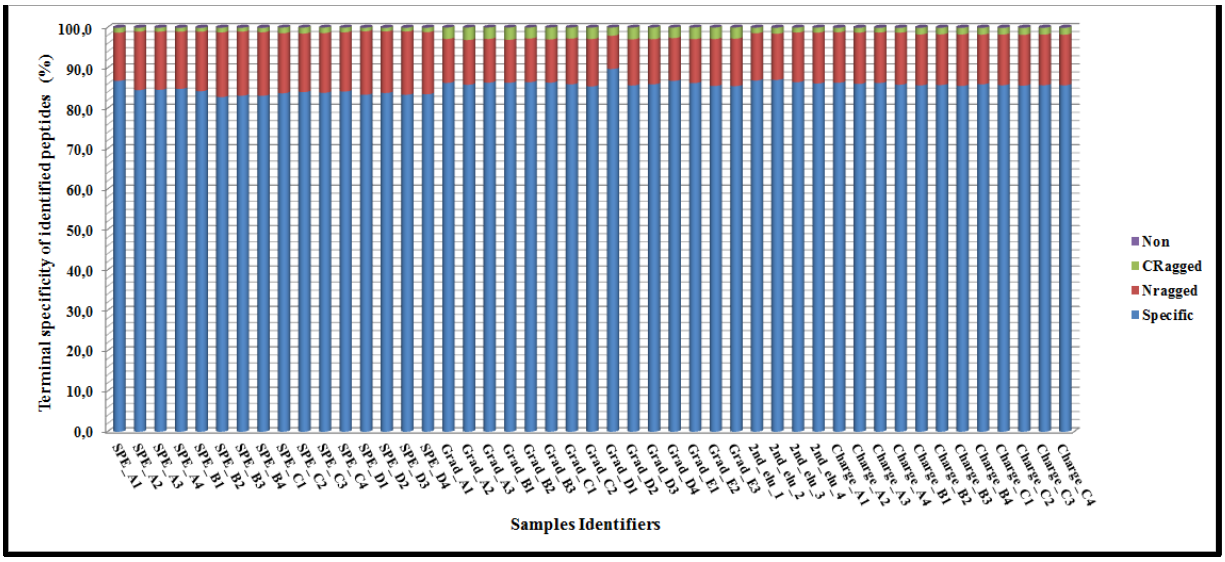

Figure S5. The optimized cell lysis, membrane preparation and solubilization processes resulted the here presented distributions of peptides terminal specificity. Identifiers of the 47 mass spectrometry runs are on the horizontal axis, while the percentage distributions of peptid terminal specificity are on the vertical axis. Terminal specificity labeled as follows: Non: non tryptic terminals; Cragged: C-terminal non-specific; Nragged: N-terminal non-specific; Specific: tryptic terminals (Abbreviations: SPE: solid-phase extraction; Grad: gradient; 2nd elu: second elution; Charge: charge state)

Figure S6. Dot blot analysis for the verification that the biotinylated components remain on the affinity column

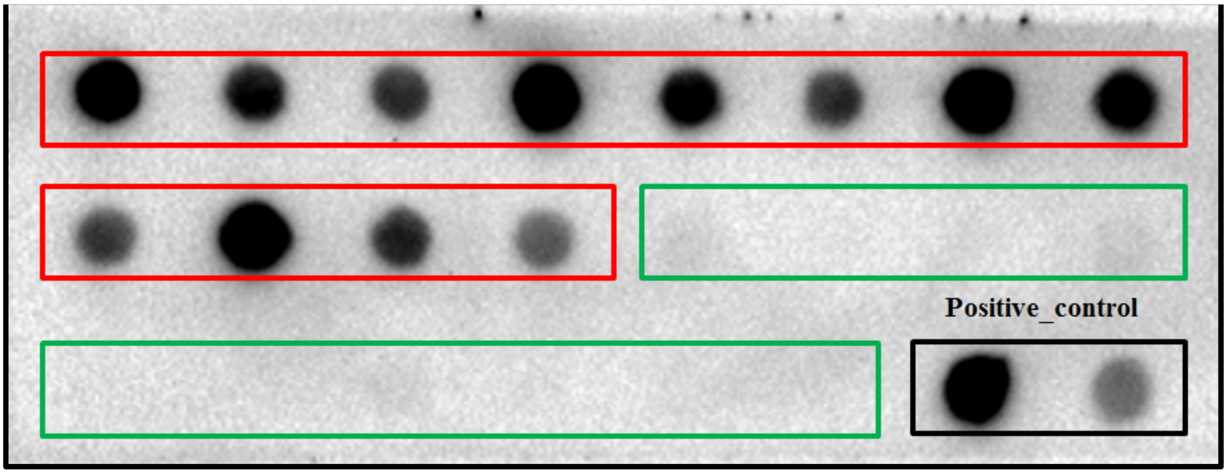

Figure S6. Dot blot measurement to indicate that all of the labeled peptides are immobilized on the column. In the red frame are the before column fractions and in the green frame are the after column or flow through fractions (three spots belong to one group from left to right in ½ dilution series, the positive control is biotinylated BSA).

Figure S7. SDS-PAGE analysis to verify the usefulness of the second elution

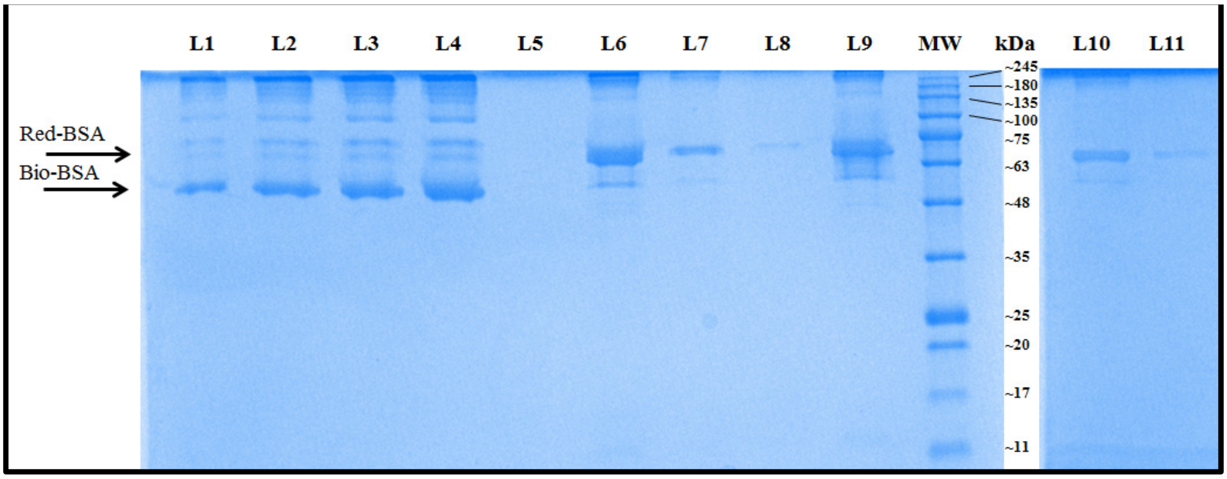

Figure S7. SDS-PAGE analysis to test the multi-step elution of the biotinylated bovine serum albumin from the affinity column. L1-L2-L3-L4 are biotinylated BSA (protein contents are 1, 2, 3 and 4 µg, respectively) from the before column fraction, L5 is the flow through fraction (indicate that the BSA stayed on the affinity column), while L6-L9; L7-L10 and L8-L11 are the first, the second and the third fractions after the DTT elution. MW: GRS Protein Marker Multicolour, Red-BSA: reduced BSA, Bio-BSA: biotinylated BSA. The gel was captured by a Bio-Rad ChemiDoc XRS+ Imaging system.

Figure S8. Results of the HPLC gradient optimization

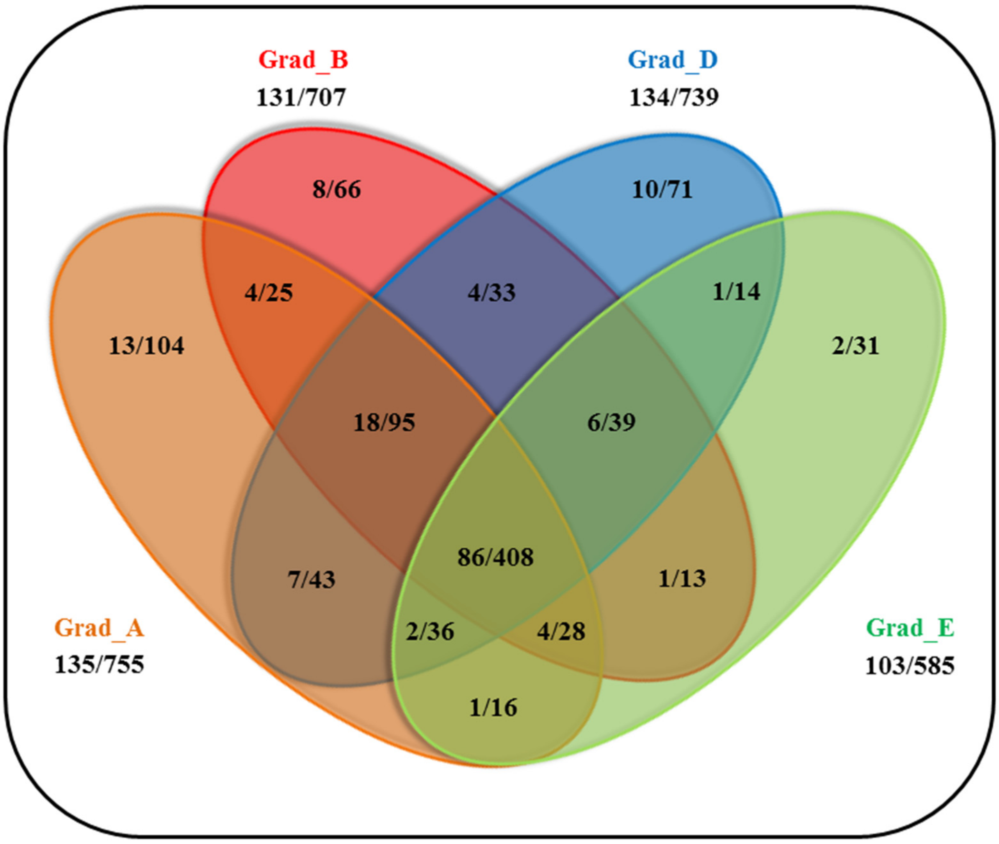

Figure S8. Venn diagram showing the number of individually labeled CSPs/ the number of their labeled peptides that were identified by using different HPLC gradients.

Figure S9. Results of the various precursor charge preferences experiments

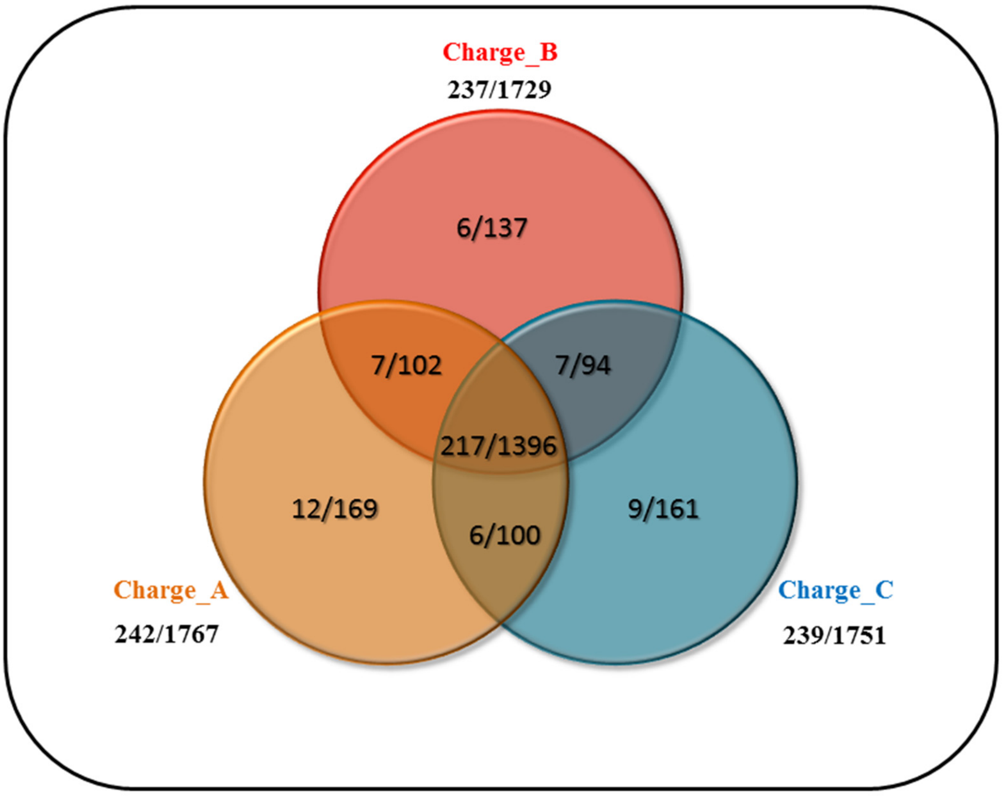

Figure S9. Venn diagram showing the number of individually labeled CSPs/ the number of their labeled peptides that were identified by using different precursor charge state preferences under the tandem mass spectrometry analysis.

Figure S10. Original dot blot to Figure 2A.

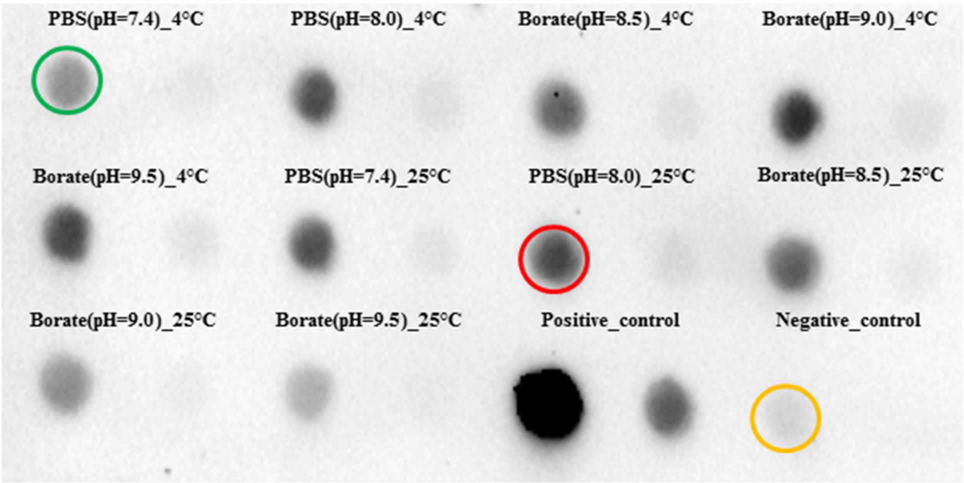

Figure S10. HL60 cells were labeled in different buffer conditions (indicated above the appropriate spot) during the same time intervals and biotin content of their membrane preparations were analyzed by dot blot (positive control: biotinylated BSA, negative control: non-biotinylated membrane preparations). The result of "PBS (pH=7.4)_4°C condition" is indicated by a green circle, while "PBS (pH=8.0)_25°C condition" is labeled by red, and negative control is an orange circle.

Figure S11. Complementary SDS-PAGE to the Western blot analysis of Figure 2C

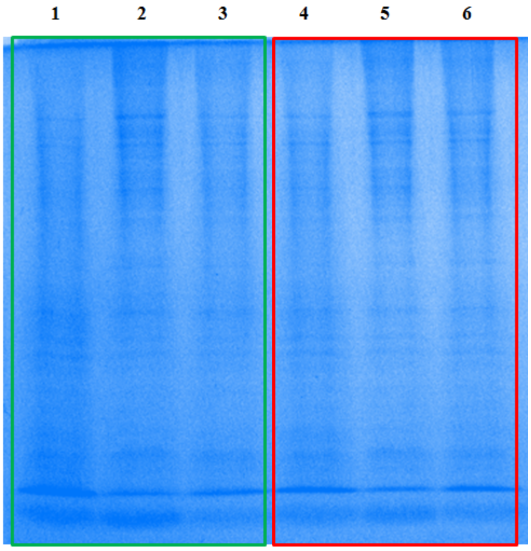

Figure S11. Complementary SDS-PAGE to the Western blot analysis of Figure 2C. 1-3: HL60 biotinylated (pH=7.4; 4°C) membrane preparations; 4-6: HL60 biotinylated (pH=8.0; 25°C) membrane preparations. The gel was captured by a Bio-Rad ChemiDoc XRS+ Imaging system.

Figure S12. Biotinylated HL60 cell surfaces were analyzed by confocal microscopy and flow cytometry measurement

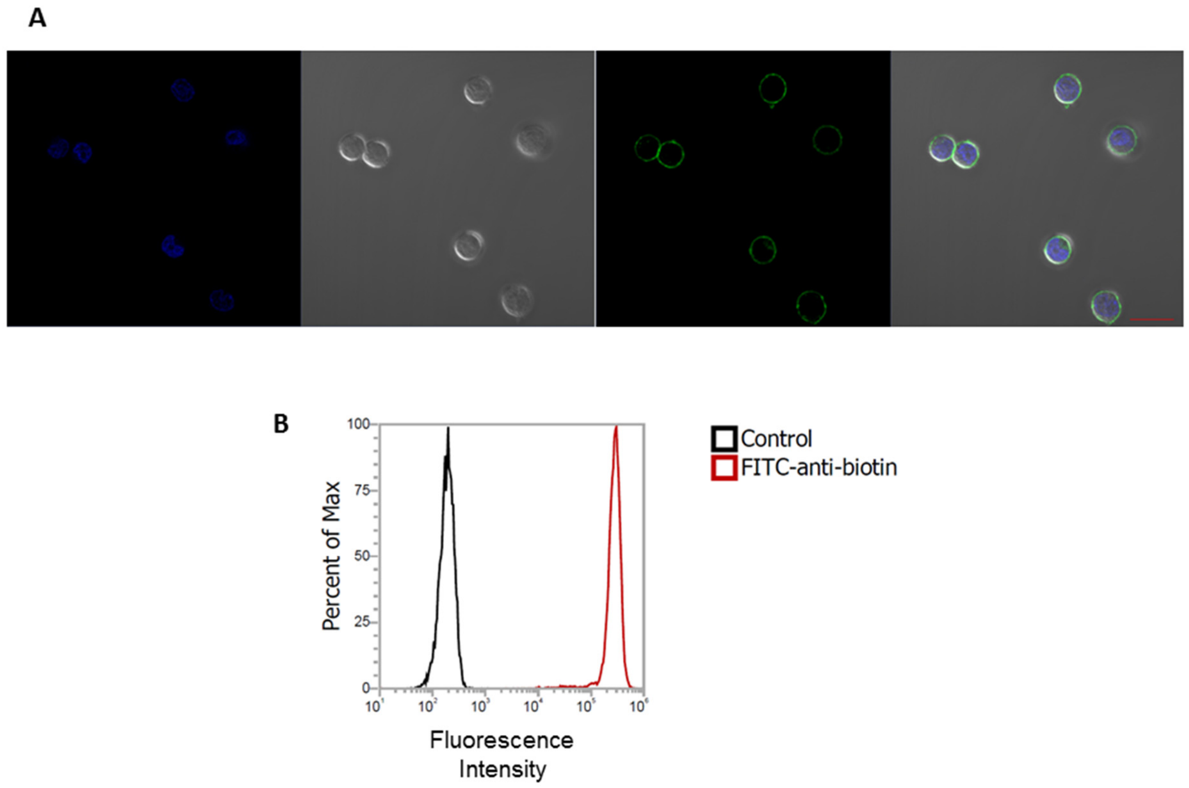

Figure S12. A) Biotinylated HL60 cells were treated with FITC-conjugated anti-biotin and were analyzed with confocal microscopy (from left to right: Hoechst 33342 DNA dye fluorescence, DIC, Labeled extracellular surface (FITC-anti-biotin), Merge). Red scale bar: 20 µm. Zeiss ZEN lite software (Carl Zeiss, Oberkochen, Germany) was used to acquire images. B) Unbiotinylated (control) and biotinylated cells were monitored by flow cytometry using FITC-conjugated anti-biotin antibody.

Figure S13. CD45 protein surface localization and CD45-biotinylation co-localization on HL60 cell surfaces were analyzed by confocal microscopy and flow cytometry measurement

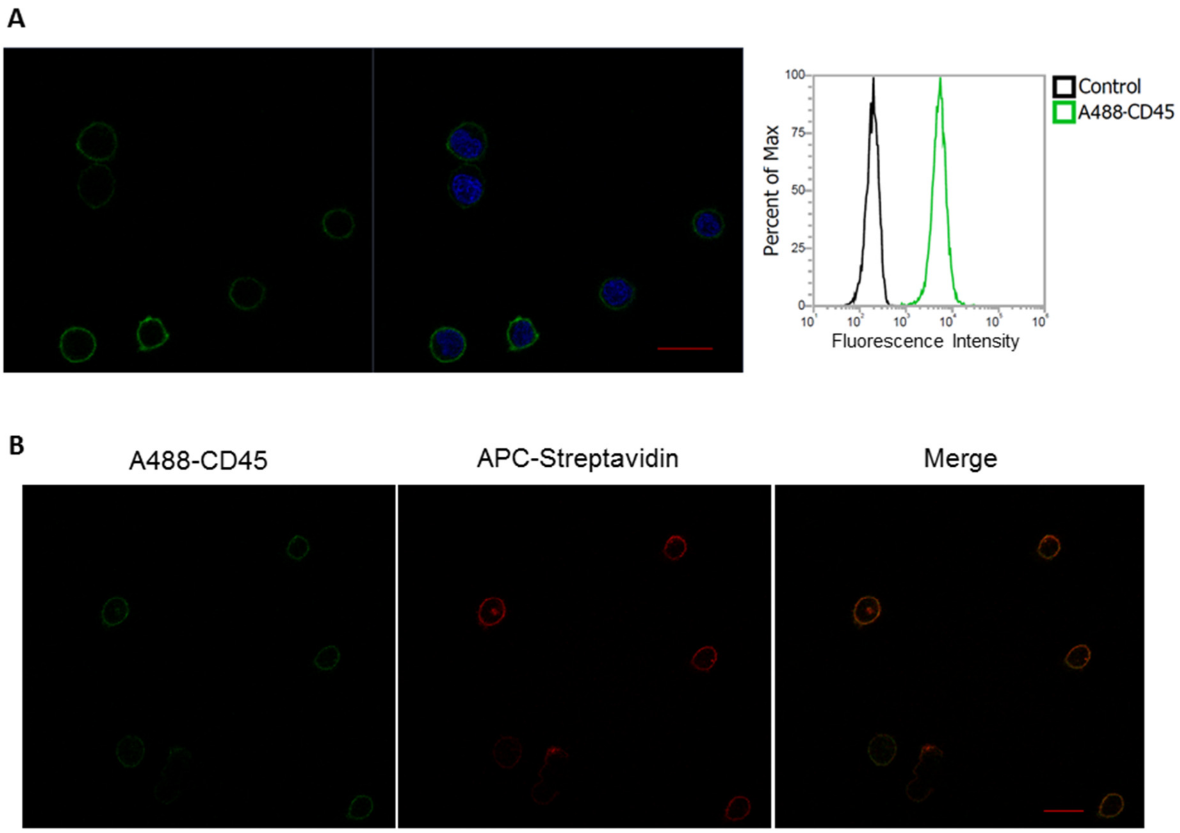

Figure S13. A) Surface localization of CD45 was analyzed by Alexa Fluor 488-conjugated CD45 antibody and detected with confocal microscopy and flow cytometry (left to right A488-conjugated-CD45 antibody staining then it was merged Hoechst33342 DNA dye, and flow cytometry measurement). Red scale bar: 20 μm. B) CD45 and cell surface biotinylation co-localization by confocal microscopy. Red scale bar: 20 μm. Zeiss ZEN lite software (Carl Zeiss, Oberkochen, Germany) was used to acquire images.

Figure S14. Original Western blot to Figure 2C.

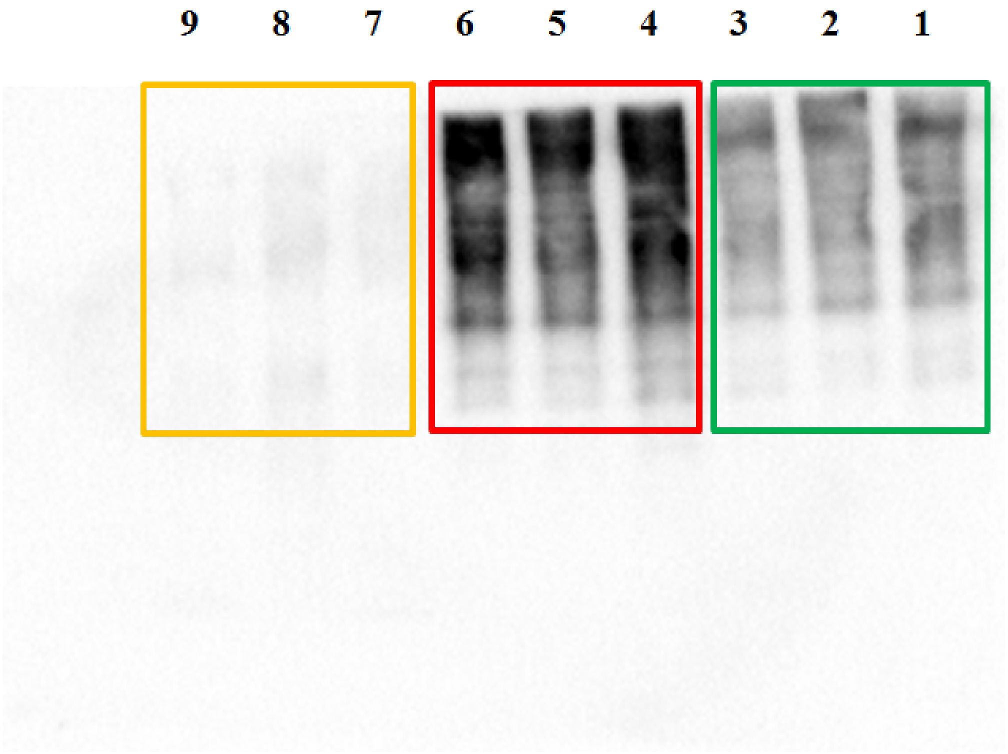

Figure S14. Original Western blot to Figure 2C. Western blot analysis to confirm the effectiveness of the labeling (colors indicate the type of the sample as Figure 2C). 1–3: HL60 biotinylated (pH = 7.4; 4 °C) membrane preparations; 4–6: HL60 biotinylated (pH = 8.0; 25 °C) membrane preparations; 7–9: HL60 non-biotinylated membrane preparations.

Supplementary Tables

Table S1. Steps of the four different SPE methods for peptide purification. Abbreviations in the Table: MeOH: methanol, ACN: acetonitrile, TFA: trifluoroacetic acid, HFBA: heptafluorobutyric acid, H_3PO_4: phosphoric acid, FA: formic acid; all reagents were obtained from Sigma-Aldrich.

**Protocol**	**General C_{18}**	**Hydrophil-optimized C_{18}**	**Oasis-HLB**	**TopTip**
**Stationary phase**	C_{18}	C_{18}	HLB	Graphite + C_{18}
**Step 1. Activation**	200 µL 50% MeOH, twice	200 µL 50% MeOH, twice	100 µL MeOH, twice	100 µL 80% ACN 0.1% TFA, twice
**Step 2. Equilibration I**	200 µL 0.5% TFA 5% ACN, twice	200 µL 0.5% TFA 5% ACN, twice	100 µL water, twice	100 µL water, twice
**Step 3. Equilibration II**	200 µL 0.1% TFA, twice, then the cartridge was transferred to a new tube	200 µL 0.1% HFBA, twice, then the cartridge was transferred to a new tube	100 µL water, then the cartridge was transferred to a new tube	100 µL water, then the cartridge was transferred to a new tube
**Step 4. Dissolving of the dried peptide samples and application on column**	50 µL 0.1% TFA, then apply the flow through once more	50 µL 0.1% HFBA, then apply the flow through once more	50 µL 2% H_3PO_4, then apply the flow through once more	50 µL water, then apply the flow through once more
**Step 5. Wash**	100 µL 0.1% TFA, twice, then the cartridge was transferred to a new tube	100 µL 0.1% HFBA, twice, then the cartridge was transferred to a new tube	50 µL 5% MeOH, thrice, then the cartridge was transferred to a new tube	50 µL water, thrice, then the cartridge was transferred to a new tube
**Step 6. Elution I**	50 µL 70% ACN 0.1% TFA, twice	50 µL 70% ACN 0.1% TFA, twice	50 µL MeOH, thrice	50 µL 40% ACN 0.05% TFA, thrice
**Step 7. Elution II**	-	50 µL 70% ACN 0.1% FA, once	-	-
**Centrifugation between steps**	2000 g, 1 min	2500 g, 1 min	3000 g, 1 min	3000 g, 1 min

Table S2. Five different gradients for the HPLC peptide separation.

Time point	Grad_1 B%	Grad_2 B%	Grad_3 B%	Grad_4 B%	Grad_5 B%
0	4	4	4	10	10
8	4	4	4	10	10
98		45	20	45	
128	50	50	50	50	50
130	90	90	90	90	90
133	4	4	4	10	10

Table S3. Comparison of the present and other published cell surface peptide isolation protocols

	Present study	Our previous work[19]	CSPA[20]
**Required cell number**	~10^6	~10^7	~10^8
**Targeted region frequency**	Frequent	Frequent	Less frequent
**Physiological labeling condition**	Yes	Yes	No
**Optimization of labeling condition at temperature- and pH- level**	Yes	No	No
**Endogenous proteases elimination**	Yes	No	Yes
**Elution optimization from the affinity column**	Yes	No	No
**SPE, HPLC and MS optimization for the labeled segment identification**	Yes	No	No
**The size of the detected modification**	+88 and 145 Da	+88 and 145 Da	+1 Da
**Modified peptide identification (/MS run)**	High	Low	Medium
**Cell surface protein identification**	More comprehensive	Comprehensive	Comprehensive
**Topologically correct labeling**	High	High	Medium
**Number of extracellular segment/protein**	High	Low	Low

Tables are presented as separate xlsx files

Table S4. Byonic search engine parameters and list of all detected peptides for second elution and SPE experiments from HL60 cells

Search engine parameters are presented in the Byonic_search_parameters sheet. Surface-exposed peptides are listed on separate sheets for second elution and SPE experiments.

Table S5. List of all detected peptides for HPLC gradient optimization and precursor charge state experiments from HL60 cells

Surface-exposed peptides are listed on separate sheets for HPLC gradient optimization (Grad sheet) and precursor charge state (Charge sheet) experiments.

Table S6. List of all labeled peptides and classification of modified proteins

The peptides carrying the artificial modifications by our labeling process were listed here from all nanoHPLC-MS/MS runs, and the proteins containing them classified based on CCTOP algorithm and UniProt annotations as described in Assessment of identified proteins and peptides section.

Table S7. List of the labeled positions of TMPs and their topological validation

The labeled positions from TMPs are presented here. The number of their detections, their topological verifications and the TmAlphaFold evaluation of the proteins are in the second, third and fourth columns, respectively (those proteins that could have originated from intracellular compartments are highlighted with grey background). Each labeled position in the first column and the TmAlphaFold evaluation result in the fourth column are hyperlinked to the TmAlphaFold database, where the predicted topology and 3D structure of the labeled proteins can be found and the atoms of the labeled residues are shown with cyan balls.
